# Triplet–Triplet Annihilation Upconverting Liposomes: Mechanistic Insights into the Role of Membranes in Two-Dimensional TTA-UC

**DOI:** 10.1021/acsami.4c00990

**Published:** 2024-05-22

**Authors:** Amrutha Prabhakaran, Keshav Kumar Jha, Rengel Cane E. Sia, Ruben Arturo Arellano Reyes, Nirod Kumar Sarangi, Mateusz Kogut, Julien Guthmuller, Jacek Czub, Benjamin Dietzek-Ivanšić, Tia E. Keyes

**Affiliations:** † School of Chemical Sciences and National Centre for Sensor Research, Dublin City University, Dublin 9, Ireland; ‡ Research Department Functional Interfaces, Leibniz Institute of Photonic Technology Jena, Jena 07745, Germany; § Institute of Physical Chemistry and Abbe Center of Photonics, Friedrich Schiller University Jena, Jena 07743, Germany; ∥ Institute of Physics and Applied Computer Science, Faculty of Applied Physics and Mathematics, Gdańsk University of Technology, Narutowicza 11/12, 80233 Gdańsk, Poland; ⊥ Department of Physical Chemistry, Gdańsk University of Technology, Narutowicza 11/12, 80233 Gdańsk, Poland

**Keywords:** triplet−triplet annihilation upconversion (TTA-UC), BODIPY, intersystem crossing (ISC), large unilamellar vesicles (LUVs), giant unilamellar vesicles (GUVs)

## Abstract

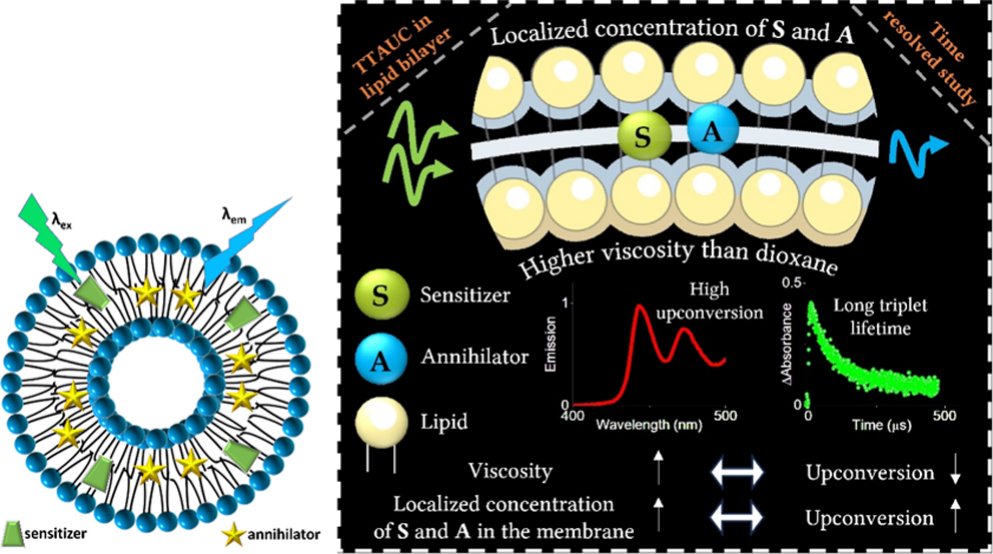

Triplet–triplet annihilation upconversion (TTA-UC) implemented in nanoparticle assemblies is of emerging interest in biomedical applications, including in drug delivery and imaging. As it is a bimolecular process, ensuring sufficient mobility of the sensitizer and annihilator to facilitate effective collision in the nanoparticle is key. Liposomes can provide the benefits of two-dimensional confinement and condensed concentration of the sensitizer and annihilator along with superior fluidity compared to other nanoparticle assemblies. They are also biocompatible and widely applied across drug delivery modalities. However, there are relatively few liposomal TTA-UC systems reported to date, so systematic studies of the influence of the liposomal environment on TTA-UC are currently lacking. Here, we report the first example of a BODIPY-based sensitizer TTA-UC system within liposomes and use this system to study TTA-UC generation and compare the relative intensity of the anti-Stokes signal for this system as a function of liposome composition and membrane fluidity. We report for the first time on time-resolved spectroscopic studies of TTA-UC in membranes. Nanosecond transient absorption data reveal the BODIPY-perylene dyad sensitizer has a long triplet lifetime in liposome with contributions from three triplet excited states, whose lifetimes are reduced upon coinclusion of the annihilator due to triplet–triplet energy transfer, to a greater extent than in solution. This indicates triplet energy transfer between the sensitizer and the annihilator is enhanced in the membrane system. Molecular dynamics simulations of the sensitizer and annihilator TTA collision complex are modeled in the membrane and confirm the co-orientation of the pair within the membrane structure and that the persistence time of the bound complex exceeds the TTA kinetics. Modeling also reliably predicted the diffusion coefficient for the sensitizer which matches closely with the experimental values from fluorescence correlation spectroscopy. The relative intensity of the TTA-UC output across nine liposomal systems of different lipid compositions was explored to examine the influence of membrane viscosity on upconversion (UC). UC showed the highest relative intensity for the most fluidic membranes and the weakest intensity for highly viscous membrane compositions, including a phase separation membrane. Overall, our study reveals that the co-orientation of the UC pair within the membrane is crucial for effective TTA-UC within a biomembrane and that the intensity of the TTA-UC output can be tuned in liposomal nanoparticles by modifying the phase and fluidity of the liposome. These new insights will aid in the design of liposomal TTA-UC systems for biomedical applications.

## 1 Introduction

Triplet–triplet annihilation upconversion (TTA-UC) is a photophysical process in which two low-energy excitation photons are combined through a bimolecular process to yield the emission of a high-energy photon.^1−5^ While the phenomenon has been long-known,^6^ it has provoked intense scientific attention in recent years because of its potential to harness low-energy solar photons in solar cells.^7^ Additionally, since TTA-UC can potentially enable the local generation of high-energy photons inside biological tissues, it offers the prospect of initiating impenetrable UV-induced processes using tissue-penetrative red or near-infrared (NIR) excitation.^8,9^ Crucially, unlike other upconversion processes (e.g., harmonic generation),^10,11^ TTA-UC can be initiated using sensitizers with large absorbance cross sections and so is amenable to relatively low-excitation power density and noncoherent source excitation.^12−15^ TTA-UC thus holds significant potential across many application domains, from biological imaging, sensing, photodynamic therapy (PDT), and photoinduced drug release to non-biological applications that include photocatalysis, photovoltaics, and photoswitching, as well as organic synthesis.^16−19^

**Scheme 1:**
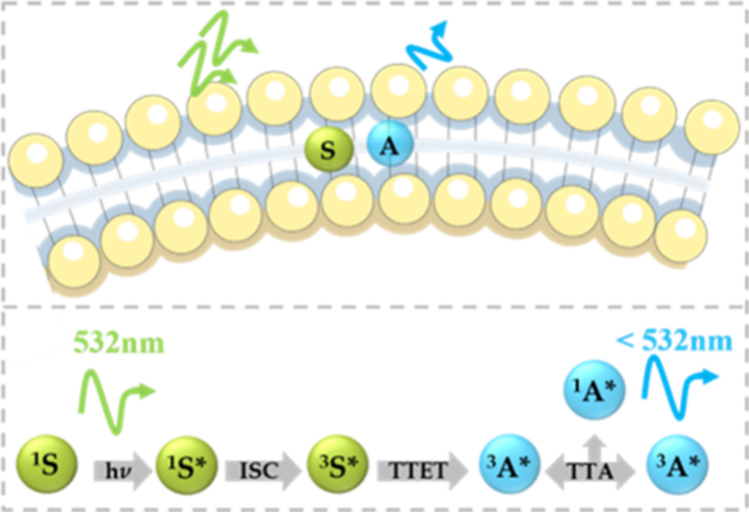
Schematic of the TTA-UC Mechanism in a Lipid Bilayer Membrane

TTA-UC is a bimolecular process. As illustrated in Scheme 1 in the lipid bilayer membrane, it requires collision between the sensitizer and the annihilator. The process is initiated by the absorption of a low-energy photon by the sensitizer (or donor) that is typically excited to its first singlet excited state (^1^S*), from where intersystem crossing (ISC) occurs to generate the triplet excited state (^3^S*). The sensitizer then undergoes triplet–triplet energy transfer (TTET) with an annihilator (A), which is excited to its first triplet excited state (^3^A*). Two triplet annihilators then undergo triplet–triplet annihilation (TTA), generating an excited singlet annihilator (^1^A*), which emits a fluorescent photon of higher energy than the initial exciting photon.^20^

A key objective in refining TTA-UC is to optimize the efficiency of the contributing TTA and UC processes by maximizing the quantum efficiency of the underlying elementary photophysical steps (ISC, TTET, and TTA) while minimizing the competing paths (triplet intrinsic decay).^22−25^

Implementation of TTA-UC in nanostructures is of significant application interest and may also be advantageous because co-location of the sensitizer and the annihilator within the nanostructure increases the local concentration and may promote Dexter energy transfer and annihilation, if issues such as aggregation do not interfere.^26−29^ Nanoencapsulation of the TTA-UC components may also provide a means to reduce the impact of oxygen on the TTA-UC process.^30,31^ An attractive way to accomplish TTA-UC is to include the pair in liposomes or micelles. These assemblies offer the aforementioned nanoscale co-constraint of reagents, with high lateral mobility to support collisional encounter between the sensitizer and the annihilator. Additionally, the lateral lipid order may inhibit aggregation depending on molecular properties. The low polarity environment of the liposome, in particular, may facilitate efficient TTA-UC by suppressing competing electron transfer reactions while facilitating TTA-UC, in aqueous media. Furthermore, both liposomes and micelles are biocompatible and widely used, for example, in imaging and drug delivery.^32,33^ Bonnet et al. employed TTA-UC as a means of imaging the membranes of giant unilamellar vesicles (GUVs) comprising DOPC or DMPC using a palladium tetraphenyltetrabenzoporphyrin (PdTPTBP) sensitizer and a perylene annihilator.^34^ Specht et al. developed light-activatable liposomes to photorelease melphalan drug using red-light TTA-UC-assisted drug photolysis with the PdTPTBP sensitizer and the *tert*-butylated perylene annihilator.^35^ Implementation of the TTA-UC components in membranes has been proposed as a route for the liposome-based delivery of the TT-UC into targeted cells and for photodynamic therapy where TTA-UC may be used for the activation of photoactive agents in the chemotherapeutic window.^36−39^ However, despite the enormous potential, there remain relatively few reports of TTA-UC in liposomal systems and none that directly address the impact of liposomal composition and viscosity on this bimolecular process.

**Figure 1:**
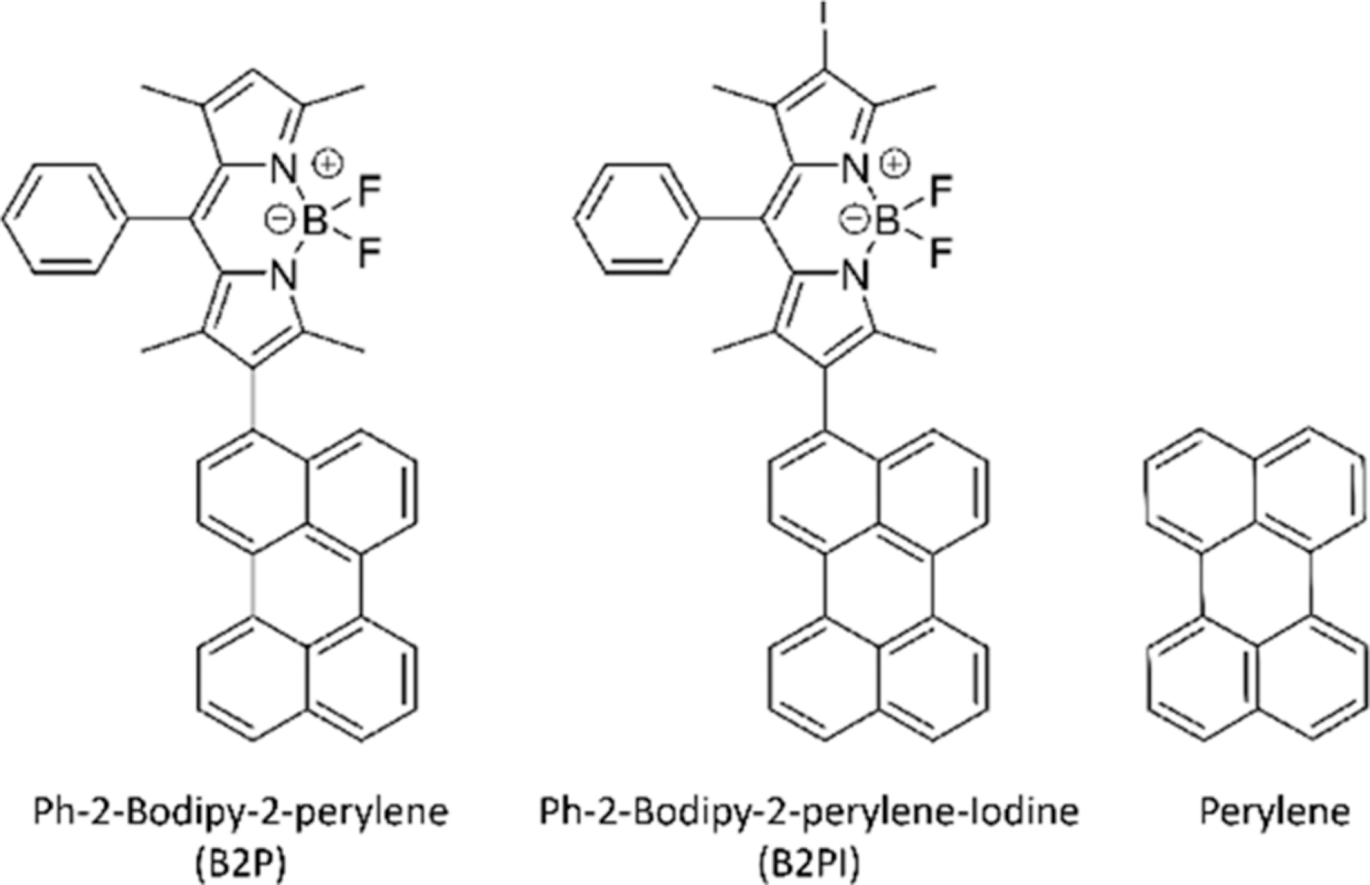
Structures of Ph-BODIPY-2-perylene (**B2P**), Ph-BODIPY-2-perylene-iodine (**B2PI)**, and perylene.

**Scheme 2:**
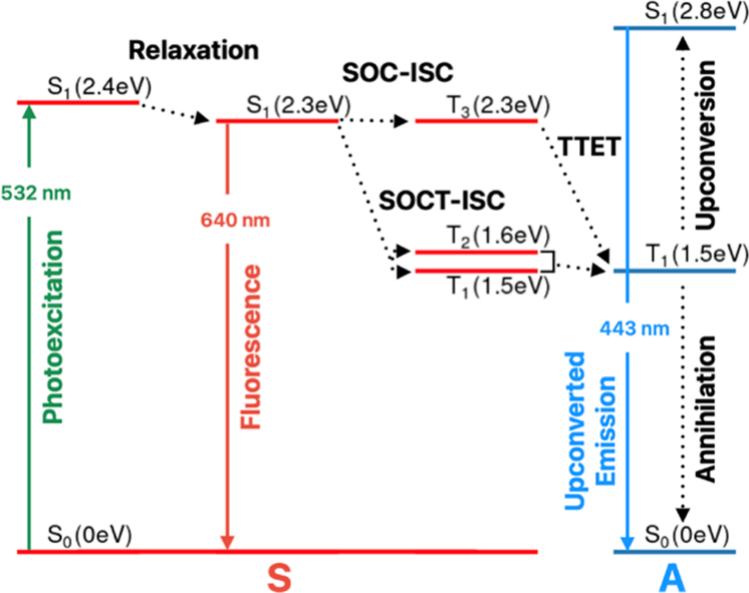
Schematic Illustration of the Jablonski Diagram of the TTA-UC Mechanism for the System Explored Here. S, Sensitizer (B2PI dyad); A, Annihilator (perylene); ISC, Intersystem Crossing; SOC-ISC, Spin–Orbit Coupling ISC; SOCT-ISC, Spin–Orbit Charge Transfer ISC; TTET, Triplet–Triplet Energy Transfer. The TDDFT (MN15/def2-TZVP) Energies of B2PI Are Indicated in Brackets^21^

A critical consideration in spatial constraint is that the sensitizer and annihilator must both localize to the same region of the liposomal structure to facilitate effective collision leading to Dexter energy transfer. To investigate this, we report the first example of all-organic, BODIPY-sensitized TTA-UC in a liposomal system using perylene as the annihilator. BODIPY derivatives are cogent sensitizers for membrane-bound systems as they are widely applied as lipid membrane probes in imaging and biophysics because of their lipophilicity and excellent photophysical properties.^40^ Additionally, while BODIPY-based donor–acceptor dyads, with enhanced triplet yield, have been used as sensitizers for TTA-UC in solution, they have not yet been applied to liposomal systems.^41^ In this work, two recently reported BODIPY-perylene dyads, with and without an iodine substitution, are applied as sensitizers (see Figure 1). These dyes were shown to exhibit spin–orbit charge transfer intersystem crossing (SOCT-ISC) as well as enhanced ISC due to the heavy atom in the iodinated derivative that promoted the formation of triplet states, leading to intense TTA-UC in solution with perylene.^4^ The mechanism and thermodynamics of this system (for the iodinated dyad, B2PI) are summarized in Scheme 2, which is derived from combined computation, and ultrafast spectroscopies reported recently in dioxane. The B2PI populates three triplet states from where TTET to the annihilator can occur. TTA-UC was found to be remarkably solvent-dependent and was observed, across 10 solvents explored, to occur only in dioxane and DMSO.^4,21^

In this report, we apply this sensitizer–annihilator system, optimized in dioxane, to liposomal systems and, supported by computation, explain why their orientation and collision in membranes support TTA. The TTA-UC system is integrated into large unilamellar vesicles (LUVs) since these are commonly applied as delivery vehicles and also into giant unilamellar vesicles (GUVs) of the same composition, to facilitate imaging. The presence and relative intensity of upconversion across membrane compositions were compared in excitation-matched systems to permit a semiquantitative comparison of the impact of the environment on TTA-UC since the determination of the quantum yield is challenging in liposomal systems. Furthermore, the impact of membrane composition on the underlying photophysical process is explored using transient spectroscopy. Overall, this study presents deep new insights into TTA-UC in the lipid bilayer and the impact of the membrane environment on the excited state and the TTA-UC process.

## 2 Results and Discussion

### 2.1 Steady-State Absorption and Emission Spectroscopy in Solution and Liposomes

The molecular structures of perylene, Ph-BODIPY-2-perylene (B2P), and Ph-BODIPY-2-perylene-iodine (B2PI) are given in Figure 1. The synthesis and spectroscopic properties of these derivatives along with their ultrafast photophysics and details of their TTA-UC in solution have been recently reported.^4,21,42^

**Figure 2:**
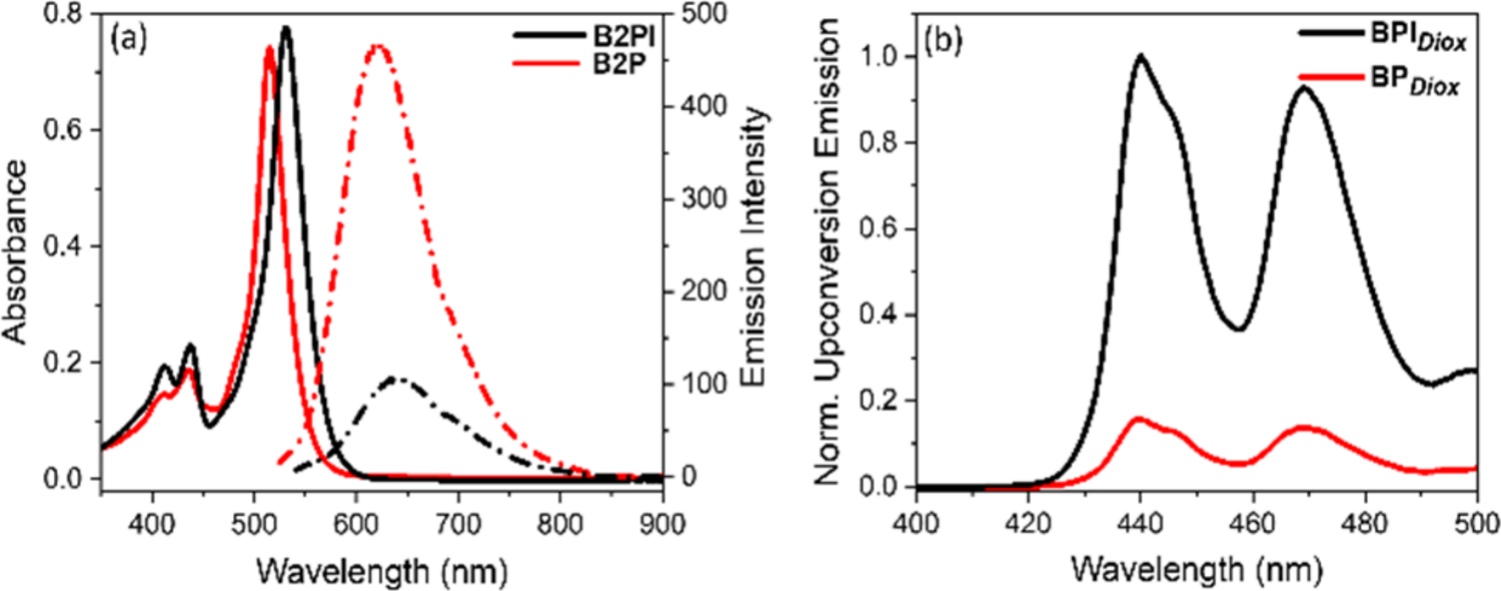
Absorption (solid line) and emission (dotted line) spectra of (a) 10 μM B2PI (black) and B2P (red) in dioxane. Emission bandwidth of B2P and B2PI is 5 nm. Emission spectra were recorded by exciting B2PI at 532 nm and B2P at 517 nm. (b) Upconversion emission from BPI_*Diox*_ (black) and BP_*Diox*_ (red); both spectra are normalized to the emission peak from the B2PI sample. The samples were excited with a 532 nm laser, and the emission was collected with 2.5 nm bandwidth. Samples were deaerated by purging N_2_.

Figure 2a shows the absorption and emission spectra of 10 μM B2P and B2PI in dioxane, selected to approximately match the dielectric with the lipid bilayer environment. Figure 2b shows the UC spectra of B2P and B2PI sensitizers with perylene in 1,4-dioxane. The absorption maximum of B2P is centered at 515 nm, while the absorption maximum of B2PI is bathochromically shifted to 530 nm on substitution with iodine. Both compounds fluoresce intensely, at 620 nm for B2P and 640 nm for B2PI, where iodination induces a red shift in the emission maxima. The fluorescence quantum yield is reduced by about 80% for B2PI. Comparable bathochromic shifts have been reported previously on the iodination of charge transfer compounds of BODIPY at the 2 and 6 positions.^1,43^

We recently reported on TTA-UC for both B2P and B2PI with perylene pairs in solution. In that study, the TTA-UC signal was optimized in dioxane where two prominent anti-Stokes emission peaks (Figure 2b) are evident at 443 and 473 nm, consistent with the fluorescence of perylene (see Figure S1). The upconversion intensity from 1 μM B2PI and 10 μM perylene in deaerated dioxane (BPI_Diox_) is approximately 10-fold higher than that from 1 μM B2P and 10 μM perylene in deaerated dioxane (BP_Diox_) (see Figure 2b) when the absorbance of both sensitizers was matched (Figure S2). The threshold power density (*I*_th_) values for BP_Diox_ and BPI_Diox_ were reported as 126 and 51 mW cm^–2^, respectively.^4^ The increased upconversion yield (ca. 8 times) and low *I*_th_ (ca. 2.5 times) for the B2PI-containing system compared to B2P, is attributable to the enhanced intersystem crossing due to the presence of iodine.^21,43−45^

TTA-UC for these pairs was found to be efficient in dioxane (dielectric constant ε_r_ = 2.25);^46^ thus, despite strong solvent dependence (where TTA-UC, across 10 solvent systems, was only observed in DMSO and dioxane), we anticipated the TTA-UC study of B2P- and B2PI–perylene pairs might also be efficient in a lipidic environment, noting that the dielectric constant is similar in DOPC (ε_r_ = 2–3).^47^

Next, we investigated whether the sensitizer can be doped into a lipid bilayer. To that end, we first incorporated B2P into DOPC LUVs of diameter ∼140 ± 5 nm (determined using DLS, cf. Figure S4, Supporting Information (SI)). In parallel, to confirm that B2P is successfully integrated and remains mobile within the lipidic environment, we performed FLIM and fluorescence lifetime correlation spectroscopy (FLCS) at pore-suspended bilayers of analogous composition. The corresponding lifetime image and autocorrelation function are provided in Figure S5 (SI). The diffusion coefficient (D) for B2P was determined as 8.6 ± 0.4 μm^2^ s^–1^ with an anomalous factor, α, value of 1.01. This value is comparable to the lipid diffusivity values reported in Table 2 (vide infra) and is consistent with membrane integration.^48^

**Figure 3:**
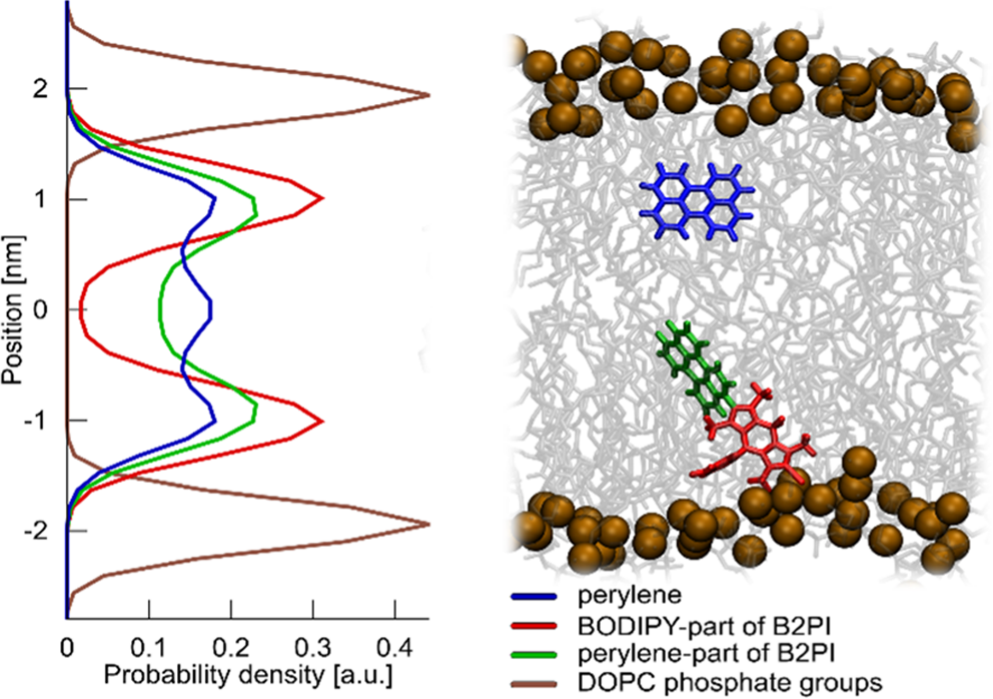
Preferred localization of the sensitizer and annihilator molecules within a DOPC membrane. The color-coded probability distributions, derived from our MD simulations, illustrate positioning along the axis perpendicular to the membrane surface. The brown curve represents the position of the DOPC phosphate groups shown for reference. The 0.0 position corresponds to the bilayer midplane.

Our molecular dynamics (MD) simulations confirm that both B2P and B2PI assemble rapidly and with high affinity into a DOPC lipid bilayer (Movie S1, link in the SI). These molecules primarily reside in the hydrocarbon core of the membrane, showing a noticeable tendency to orient their BODIPY moiety toward the polar surface while at the same time exposing their perylene moiety for interaction with the annihilator at the center of the bilayer (Figure 3). The diffusion coefficients of B2P and B2PI in the membrane, determined from our MD trajectories using mean square displacement (Figure S8), are 8.83 ± 0.68 and 8.31 ± 0.50 μm^2^ s^–1^, respectively, which align well with the measured D value for B2P.

Since TTA-UC within liposomes offers a route for upconversion in cell imaging and toward the activation wavelength of photoactivatable prodrugs closer to the phototherapeutic window,^49^ we were interested in understanding if TTA-UC could be observed in a membrane and to understand the impact of varying physicochemical properties such as fluidity/viscosity, vesicle size, hydrophobic core chain length, headgroup charge, etc. Of note, TTA-UC requires Dexter energy transfer between a photosensitizer and an annihilator; thus, in a nanoassembly, these components must co-localise to ensure that efficient molecular collision is facilitated. The lipid membrane is a suitable platform to confine the photosensitizer and annihilator to the same plane assuming both species localize to the same region of the membrane, and that their orientation is conducive to energy transfer. To that end, we first prepared large unilamellar vesicles (LUVs)^50−52^ (hydrodynamic diameter ∼140 ± 5 nm from DLS) of DOPC and two different giant unilamellar vesicles (GUVs),^53^ comprising DOPC only and a ternary phase-separated DOPC:SM (sphingomyelin):Chol (2:2:1). From imaging, GUVs were ∼10–30 μm doped with B2P- and B2PI–perylene pairs.

**Figure 4:**
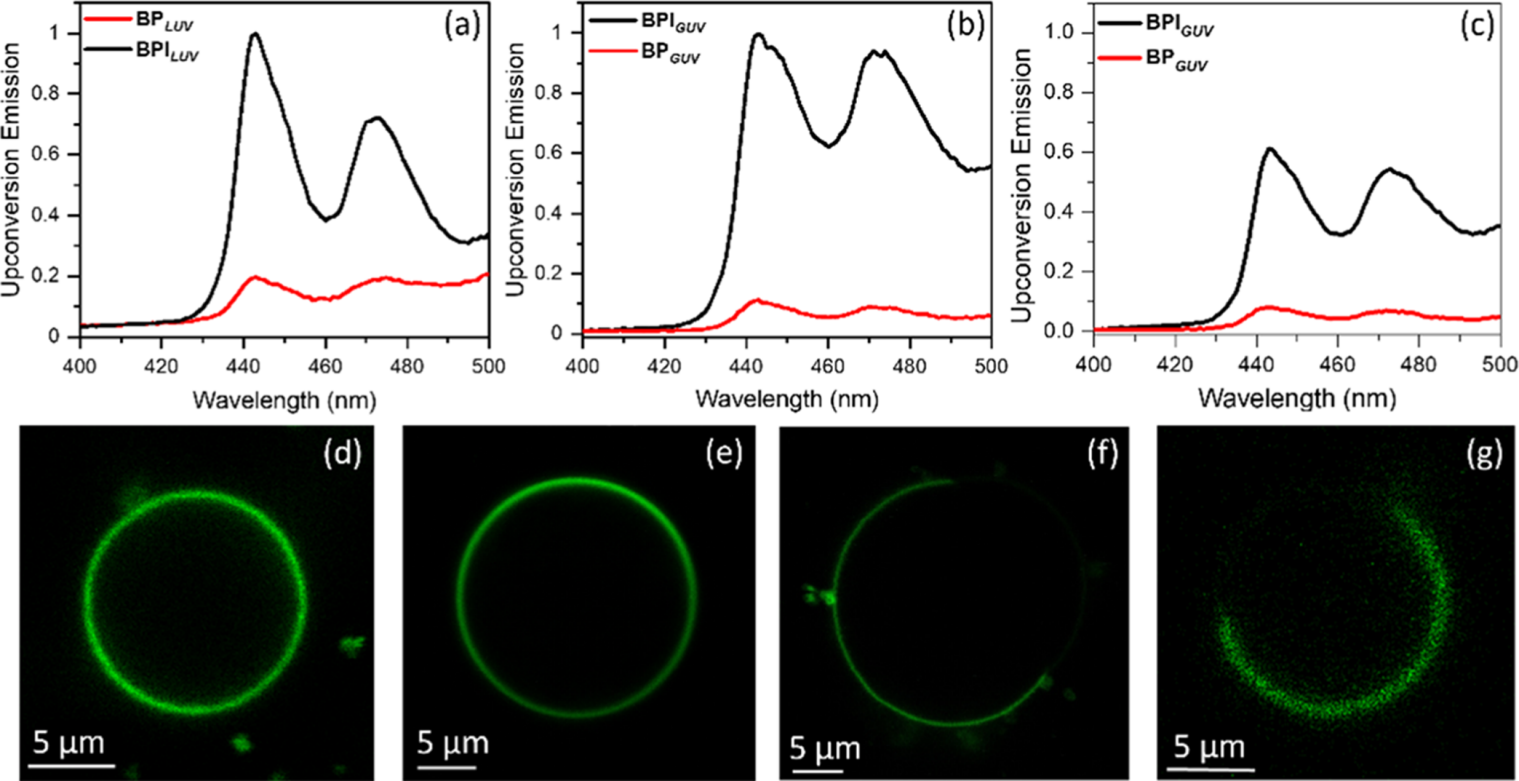
Normalized TTA-UC emission from (a) BP_LUV_ and BPI_LUV_ in DOPC liposomes of ∼140 nm size; BPGUV and BPIGUV in giant unilamellar vesicles of (b) DOPC lipid, and (c) mixture of DOPC, SM, and cholesterol (2:2:1). The upconverted emission in vesicles was measured in the presence of 20 mM sodium sulfite at a 2.5 nm emission slit width. The samples were excited with a 532 nm laser of 10 mW power. Confocal microscopy images of DOPC (d, e) and DOPC:SM:Chol (f, g) GUVs showing the membrane doped with B2P (d, f) and B2PI (e and g). The concentration of both B2PI and B2P in GUVs was ∼0.38 μM. The scale bar in panels d–g is 5 μm, and λ_ex_/λ_em_ = 514/600–700 nm. The normalization of c with respect to the maximum intensity of b is for comparison.

To investigate TTA-UC in DOPC LUVs, we compared BPI_LUV_ (doped with 0.25 μM B2PI and 2.5 μM perylene) and BP_*LUV*_ (doped with 0.25 μM B2P and 2.5 μM perylene). The concentrations of the sensitizer and the annihilator quoted are those applied before extrusion (but are assumed fully integrated into the LUV on the basis of the absence of a residual compound on the extrusion filter or filtrate). TTA-UC from LUVs were studied in a nitrogen-saturated solution in the presence of 20 mM sodium sulfite as an oxygen scavenger. Both LUV assembled pairs showed substantial anti-Stokes shifted emission, as shown in Figure 4a, attributed to TTA-UC. Comparing BPI_LUV_ and BP_*LUV*_ under identical conditions (the sensitizer is absorbance-matched at the excitation wavelength and the excitation power is identical) enabled us to compare the intensity directly, in the absence of quantum yield, and we observed that BPI_LUV_ showed a dramatically enhanced upconversion emission intensity compared to BP_*LUV*_ (Figure 4a). The intensity was greatly enhanced compared to the solution TTA-UC, to the extent that the concentration of the annihilator and the sensitizer had to be diluted 4-fold to avoid detector saturation using the same optical system for detection. With this 4-fold dilution, the intensity of the upconverted signal from the B2PI–perylene pair was reduced by only approximately 50% (Figure S3) compared to the solution. The data suggests that the B2PI–perylene pair works well in the liposome formulation because its localization and orientation in the membrane facilitate TTA- UC.

**Figure 5:**
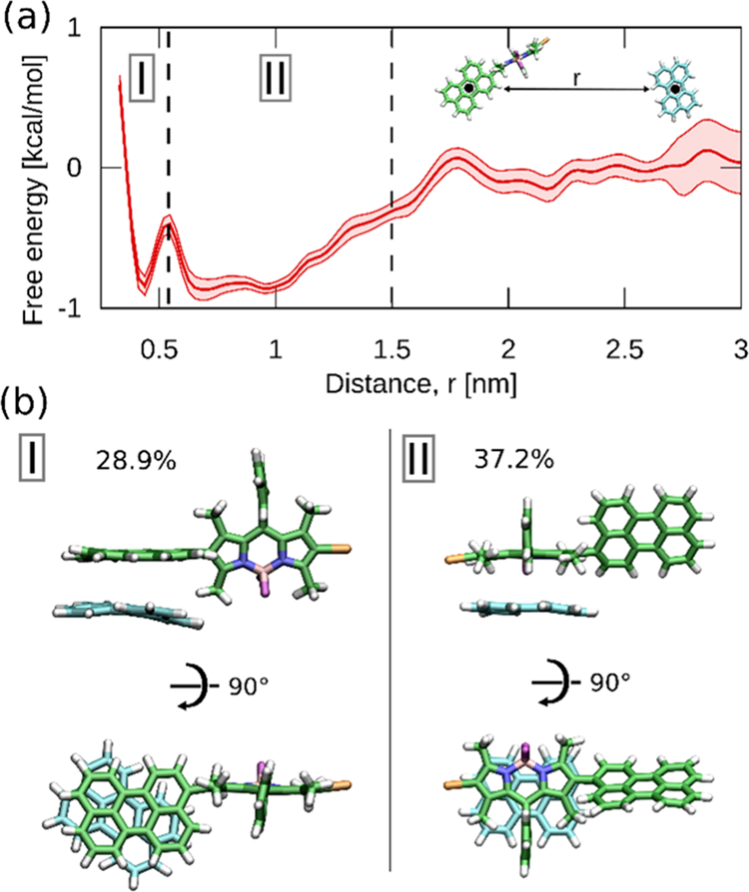
(a) MD-derived free energy profile for the B2PI–perylene interaction in a DOPC lipid bilayer. The two indicated minima, I and II, correspond to the two types of collision complexes identified in the MD simulation. (b) The most representative structures of the B2PI/perylene collision complexes of types I and II along with their relative populations in the bound state. For the sake of clarity, the B2PI and perylene carbon atoms are colored green and cyan, respectively. For full structural analysis of the identified complexes, see Figure S10.

To understand this further and provide molecular-level description of the interaction between B2PI and perylene within the membrane, we employed umbrella sampling MD simulations^54,55^ to determine the free energy profile for B2PI–perylene binding in a DOPC lipid bilayer (see Methods, SI). The resulting profile in Figure 5a reveals that B2PI and perylene interact to form several different types of collision complex, characterized by shallow free energy minima. In the most tightly bound complex (*r* < 0.55 nm I in Figure 5a), the annihilator perylene molecule orients itself parallel to the perylene moiety of B2PI in a way that should facilitate efficient triplet–triplet energy transfer (I in Figure 5b). In contrast, the broader free energy minimum (0.55 < *r* < 1.5 nm, II in Figure 5a) corresponds to multiple more loosely bound complexes in which perylene interacts mostly with the BODIPY moiety (II in Figure 5b). At the local concentration of perylene in the bilayer used in the simulation (∼20 mM), the fraction of B2PI that at any given moment remains bound in the type I complex is 0.64%. The low free energy barrier to dissociation of ∼0.5 kcal/mol observed in Figure 5, along with the additional MD simulations of spontaneous binding/dissociation (Movie S2, link in the SI), demonstrates the short-lived nature of tightly bound B2PI/perylene complexes, which have an average lifetime of 7.02 ± 0.43 ns. However, this duration is still sufficient time for the TTET process, which occurs within a few nanoseconds. Notably, the computed binding properties of both B2P and B2PI with perylene are similar (Figure S10), indicating that the higher relative intensity of TTA-UC observed for the latter originates from the more efficient intersystem crossing in the iodinated compound.^4^

To further investigate the effect of the liposome environment on the TTA-UC process, cell-sized, single-component DOPC and phase-separated DOPC:SM:Chol (2:2:1) GUVs were prepared with the sensitizer–annihilator pair to permit direct visualization of the emission from the sensitizer using confocal fluorescence microscopy. Here, the B2PI-containing compositions are referred to as BPI_GUV_ and B2P as BP_GUV_. From the confocal fluorescence images shown in Figure 4d–g, and consistent with the theory, both BPI_GUV_ and BP_GUV_ are integrated into the membrane, providing clear images of the GUVs. In the ternary composition, the probe partitions to the liquid disordered (*L*_d_) phase, confirmed by colabeling with an *L*_d_ partitioning probe DiD (DiIC18(5) solid (1,1′-dioctadecyl-3,3,3′,3′-tetramethylindodicarbocyanine, 4-chlorobenzenesulfonate salt)) as shown in Figure S11 (SI).

Figure 4b,c shows the comparative TTA-UC emission intensity for BPI_GUV_ (∼0.38 μM B2PI and ∼3.8 μM perylene in deaerated GUV) and BP_GUV_ (∼0.38 μM B2P and ∼3.8 μM perylene in deaerated GUV) in DOPC and DOPC:SM:Chol GUVs. Although the trend of BPI_GUV_ emission intensity is higher than that of BP_*GUV*_ in both of the GUVs, when compared, the overall intensities are modestly decreased for DOPC:SM:Chol compared to DOPC. This decrease may, in part, be due to the higher viscosity of the DOPC:SM:Chol membrane compared to DOPC. However, as shown in Figure S11, unlike BODIPY, perylene does not show phase selectivity and emits from both *L*_d_ and *L*_o_ phases; thus, the local concentration of perylene within the *L*_d_ phase where the sensitizer resides is reduced, reducing collision probability and leading to this observed decrease in TTA-UC.

### 2.2 Time-Resolved TTA-UC in Liposomes

**Figure 6:**
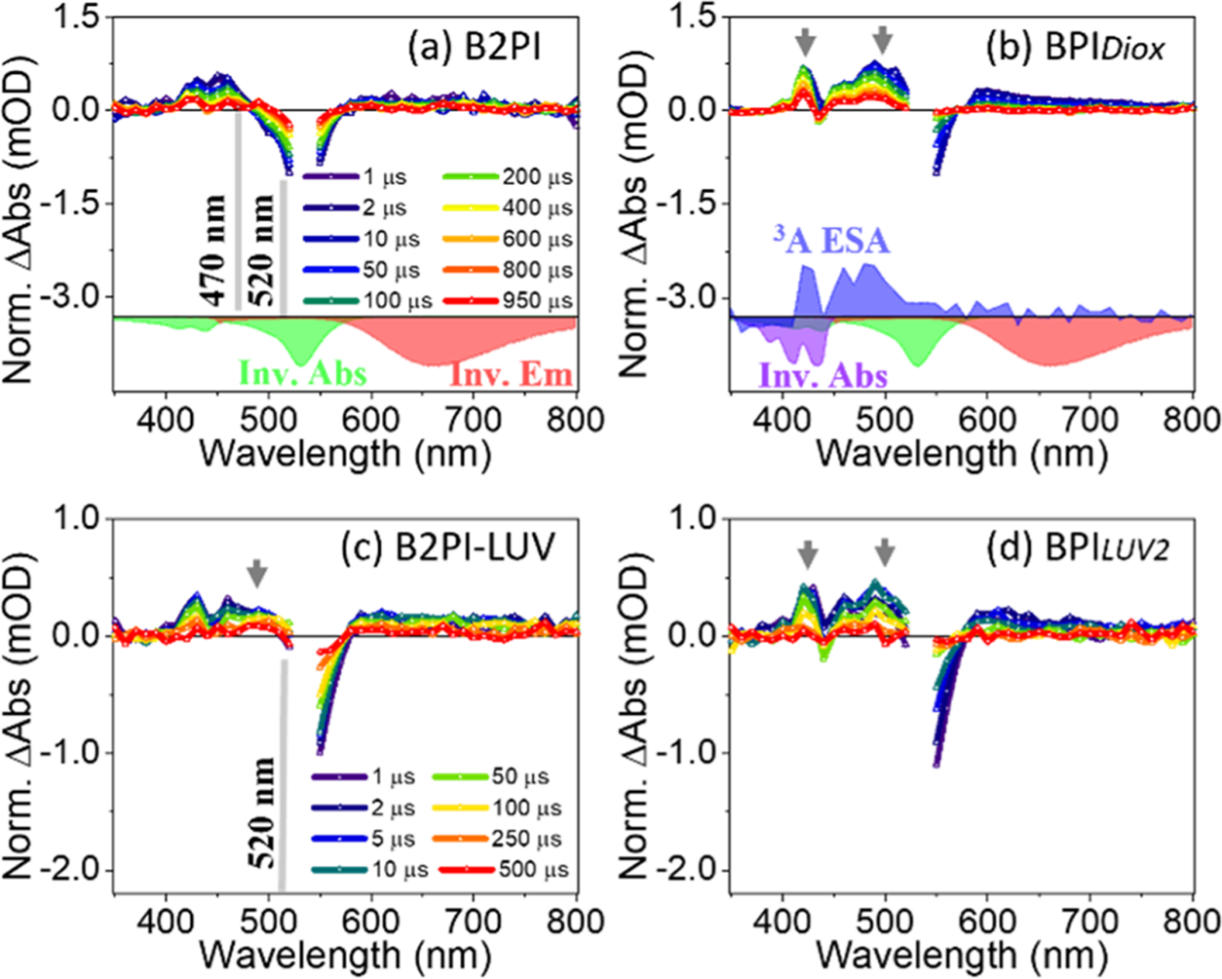
Nanosecond transient absorption spectra of (a) B2PI—1 μM in dioxane, (b) B2PI—1 μM and perylene and 10 μM in dioxane: BPI_Diox_; (c) B2PI—1 μM in LUV, (d) B2PI—1 μM and perylene—10 μM in LUV: BPI_*LUV(2)*_. Arrows in (a)–(b) and (c)–(b) show changes in the spectra with respect to each other upon addition of 10 μM perylene. (a) and (b) have the same legends; similarly, (c) and (d) have the same legends. In (a) and (b), inverted absorption and emission of B2PI are shown as green and red curves, respectively; the inverted absorption of perylene is in violet. The samples were excited with a 532 nm laser. The 530 and 540 nm kinetics are not measured due to high scattering from LUVs.

Next, to understand the impact the lipid bilayer has on the dynamics of the BODIPY triplet state, nanosecond time-resolved (ns-TA) spectra of B2PI were compared in dioxane and liposome. The data from dioxane are shown in Figure 6a, and the negative differential absorbance (ΔAbsorbance) signal from 470 to 570 nm is a contribution from ground-state bleach (GSB), which is also evident from its steady-state absorbance (Figure 2). Outside this region, excited-state absorbance (ESA) dominates throughout the shown spectral range. For the sample containing B2PI and perylene in dioxane (Figure 6b), in contrast to B2PI alone, ESA is dominant in the range of 470–520 nm (see arrows), which is due to TTET from B2PI to annihilator (perylene) molecules. The triplet ESA of BODIPY is known to be in the region 400–470 nm,^21^ whereas the triplet ESA of perylene is broader and observed in the region 400–570 nm.^56^ The B2PI molecule can form three triplet species upon ISC: charge transfer state triplet (T_3_), BODIPY-centered triplet (T_2_), and perylene-centered triplet (T_1_); energetically, T_3_ > T_2_ > T_1_.^4,21^ The spectra of the BODIPY-centered triplet and the perylene-centered triplet are expected to show differential absorbance features of pristine BODIPY and perylene molecules, respectively; the charge transfer state triplet is expected to have features common to both molecules.^4,21^ The intersystem crossing (ISC) from the singlet charge transfer state can progress through two pathways: spin–orbit coupling ISC leading to a triplet charge transfer state and spin–orbit charge transfer ISC leading to BODIPY- or perylene-centered states (Scheme 2).^57,58^

The ns-TA spectra of B2PI in LUV (Figure 6c) are similar to those in solution (see Figure 6a), though careful comparison reveals some important differences. The feature near 510 nm is the dominant ESA in the LUV sample, whereas GSB dominates in solution (Figure 6a). This is attributed to the confinement of hydrophobic B2PI molecules to the 2D lipid bilayer membrane. The confinement results in a much higher local concentration of the B2PI molecules (in the lipid bilayer) > 1 μM compared to the solution. This in turn increases the probability of collision, which makes intermolecular TTET from the BODIPY-centered state (T_2_) to the perylene annihilator (T_1_) more probable. Hence, a larger population of perylene-centered state formed by ISC along with TTET will exist in the membrane, leading to the enhancement of the ESA feature near 510 nm (see arrow, Figure 6c), compared with the dominant GSB of BODIPY-centered species in solution (Figure 6a).

Similarly, for the LUV sample containing B2PI and perylene, TTET from the sensitizer to the annihilator takes place, leading to enhancement of the ESA feature associated with perylene near 510 nm, as seen in Figure 6d. The high local membrane confined concentration of B2PI and perylene also facilitates TTA, and the lifetime of the three species (charge transfer state, BODIPY-centered, perylene-centered) is consequently shorter in the lipid bilayer than in solution (see Table 1). Due to the poor signal-to-noise ratio and high scattering, ns-TA of B2P molecules in the lipid bilayer could not be measured at 1 μM; increasing the B2P concentration increased the background, so these studies could not be completed.

**Table 1:** Sample Nomenclature and Lifetime Table^a^

nomenclature	constituent 1	constituent 2	dioxane/membrane	τ_1_ (μs)	τ_2_ (μs)	τ_3_ (μs)
	B2PI—1 μM		dioxane	65	530	1600
BPI_Diox_	B2PI—1 μM	perylene—10 μM	dioxane	43	80	880
BP_Diox_	B2P—1 μM	perylene—10 μM	dioxane			
	B2PI—1 μM		LUV	60	315	1500
BPI_LUV_	B2PI—0.25 μM	perylene—2.5 μM	LUV			
BPI_LUV(2)_	B2PI—1 μM	perylene—10 μM	LUV	7	48	650
BPI_GUV_	B2PI—∼0.38 μM	perylene—∼3.8 μM	GUV			
BP_GUV_	B2P—∼0.38 μM	perylene—∼3.8 μM	GUV			

a The lifetimes given are obtained using nanosecond time-resolved spectroscopy. The error margin for the lifetimes of All B2PI samples is within 10%. See the time-resolved section for details

### 2.3 Impact of Physicochemical Membrane Properties on TTA-UC

As compositions of liposome formulation vary widely and TTA-UC requires collision between the annihilator and the sensitizer, we examined the impact of physicochemical properties of the liposomes of varying compositions on TTA-UC by comparing the relative intensity of TTA-UC across different liposomes using BPI_LUV_. We first measured the fluidity in different liposomes as a consequence of varying alkyl chain lengths, degrees of saturation/unsaturation, lipid phase transition temperatures, and varied lipid headgroups and charge chemistries. The zwitterionic DOPC is diunsaturated, whose phase transition temperature, *T*_m_, is approximately −17 °C, whereas POPC is monounsaturated, with the *T*_m_ being approximately 2 °C. The zwitterionic DMPC and DLPC lipids with saturated alkyl chain lengths of C14 and C12, respectively, with *T*_m_ are, respectively, 23 and 2 °C.^59−61^ The chemical structures of individual lipids are provided in Figure S12 (SI), and the *T*_m_ values along with the headgroup/alkyl chain configuration are shown in Table 2. To obtain the lipid diffusivity in the above membrane composition, the diffusion coefficient values were measured at MSLB using FLCS (Table 2). Membranes comprising highly unsaturated phospholipids are more fluidic and more disordered than saturated lipids and are expected to accommodate a range of fluorophores including B2P and B2PI.

**Table 2:** Features of lipids used along with phase transition temperature, mathematical area of integration of the 443 nm peak, and diffusion coefficient of each lipid labeled with DOPE-Atto655 measured using FLCS. Diffusivity was measured at MSLBs as described in the SI

lipid	phase transition temp (°C)^a^*T*_m_	alkyl chain length & double bond	diffusion coefficient, D (μm^2^ s^–1^) DOPE-ATTO655	integrated area of 443 nm peak
DOPC	–17	18:1	9.6	3848
POPC	–2	16:0–18:1	8.8	1802
DLPC	–2	12:0	7.2	719
DMPC	24	14:0	3.5	43
DPPC	41	16:0	NA	128
DOPS	–11	18:1	9.5	303
DOTAP	<5	18:1	7.2	57
Egg PC			8.2	6742
bNature’s Own			7.8	6076

a Obtained from Avanti polar lipids.

b 32% DOPC, 25% DOPE, 20% cholesterol, 15% SM, and 8% PE.

**Figure 7:**
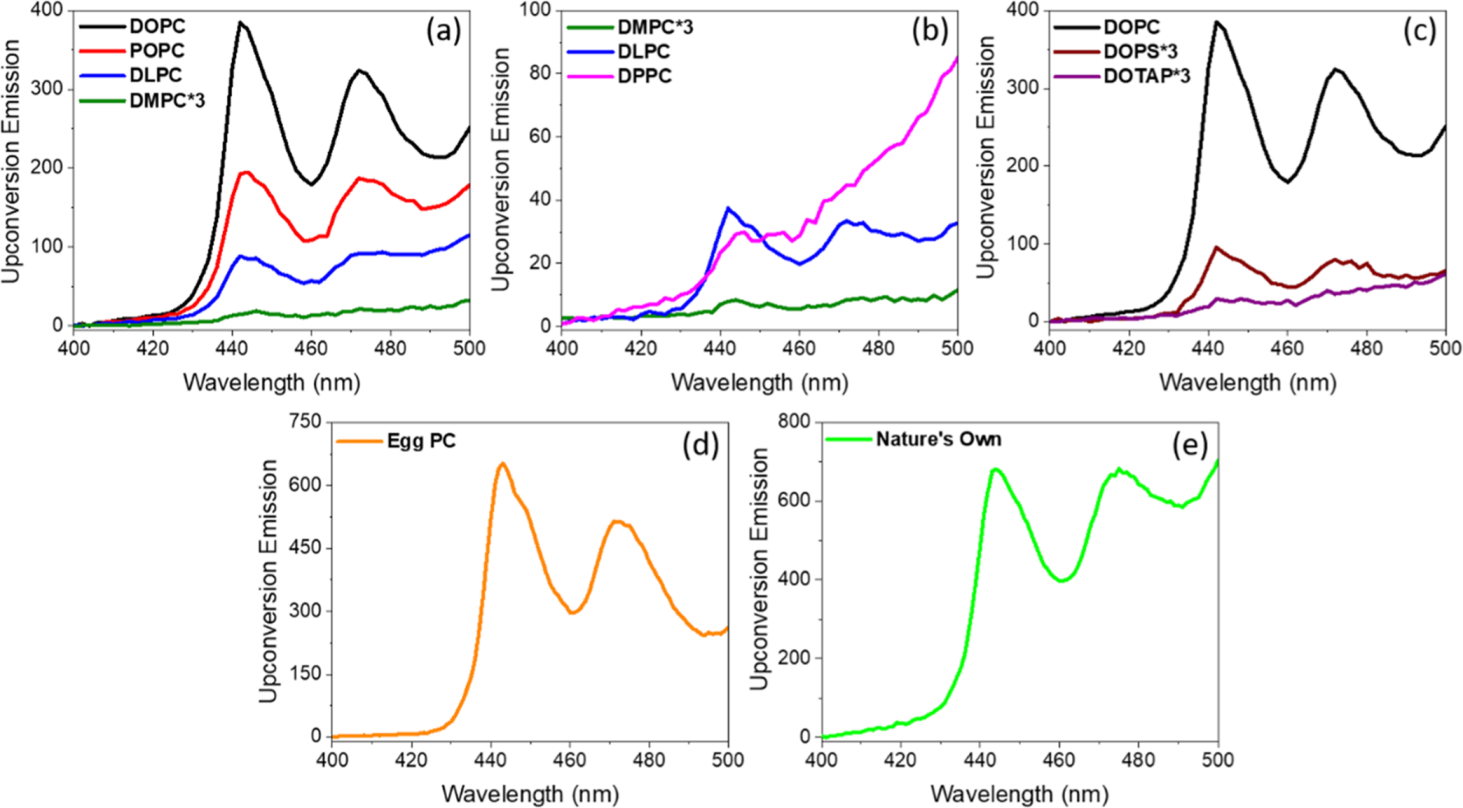
TTA-UC from liposomes of different lipids with different (a) fluidities and numbers of double bonds in DOPC, POPC, DLPC, and DMPC; (b) alkyl chain lengths in DLPC, DMPC, and DPPC; and (c) charges in DOPC, DOPS, and DOTAP. TTA-UC in (d) egg PC, and (e) mammalian plasma mimetic Nature’s Own liposomes. All samples contain 0.25 μM B2PI and 2.5 μM perylene, and the measurements were recorded at 2.5 nm emission slit width under 532 nm excitation in the presence of 20 mM sodium sulfite. All liposomes are in PBS of pH 7.4. The emission spectra of DMPC and DOTAP are multiplied by 3 for visualization and indicated in the corresponding panels by “*3”.

Figure 7a shows the upconverted emission observed from BPI_LUV_ in DOPC, POPC, DMPC, and DLPC liposomes under otherwise identical experimental conditions. We observed the following trend for the TTA relative intensity of TTA-UC DOPC > POPC > DLPC ≫ DMPC. Looking at the table of measured diffusion coefficients, this trend can largely be explained by the fluidity of the membrane. Unsurprisingly, the TTA-UC pair in DOPC liposomes showed the highest intensity upconverted signal (Figure 7a black), attributed to the high fluidity of this membrane; recalling that the diffusion coefficient of B2P was 8.6 ± 0.4 μm^2^ s^–1^, it is expected to facilitate 2D collision between the sensitizer and the annihilator, and the DOPE-ATTO655 lipid label for DOPC had a D of 9.6 ± 0.5 μm^2^ s^–1^ (DOPC melting transition temperature (*T*_m_) = −17 °C). Although they have the same *T*_m_, POPC shows greater fluidity (diffusion coefficient for the lipid label of 8.8 μm^2^ s^–1^ compared to 7.2 μm^2^ s^–1^) and DLPC. Negligible TTA-UC could be observed for DMPC membranes, consistent with the high viscosity of the DMPC membrane (*T*_m_ = 23 °C), and D (3.5 μm^2^ s^–1^) is less than half of that of DLPC, resulting in a modest TTA-UC emission. The integrated area under the peak centered at 443 nm was assessed to give some quantitative insight into the TTA-UC intensity with liposome composition and is shown as a bar plot in Figure S13. There is essentially an exponential decrease in the anti-Stokes emission intensity with increasing viscosity of the membrane, which is attributed to impeded bimolecular collision between the sensitizer and the annihilator.

To further validate this conclusion, we conducted MD simulations to estimate and compare the rate constants, *k*_on_, for the formation of the tightly bound B2PI/perylene complex (type I in Figure 5b) in the DOPC and DMPC membranes (see the SI). Our results confirmed our expectations, showing that the binding process is more than 1 order of magnitude faster in the more conformationally disordered and less viscous DOPC bilayer (*k*_on_ = 4.6 × 10^7^ M^–1^ s^–1^) than in the more ordered and densely packed DMPC bilayer (*k*_on_ = 1.2 × 10^6^ M^–1^ s^–1^). This dependence of *k*_on_ on the ordering of lipid chains can also partly be attributed to the lowering stability of the B2PI/perylene complexes with increasing lipid order revealed by our binding free energy profiles. As can be seen from Figure S14, in the DMPC bilayer, the formation of the tightly bound complex is ∼2 kcal/mol less favorable than in DOPC. Consequently, this leads to a 30-fold lower fraction of B2PI molecules bound to perylene and an elevated activation free energy ultimately leading to a decrease in the binding rate. We propose that the lower stability of the B2PI/perylene complex in membranes with a higher conformational order arises from the decreased capability of the hydrocarbon chains to accommodate bulky molecular assemblies. Another factor contributing to the lower binding rate in DMPC is the slower diffusivity, with the diffusion coefficient of B2PI in DMPC (3.552 ± 0.95 μm^2^ s^–1^) being ∼2 times smaller than that in DOPC.

Next, the impact of the alkyl chain length on TTA-UC was compared. Liposomes of three neutral membrane compositions—DLPC (C12), DMPC (C14), and DPPC (C16) liposomes—were prepared and each doped with the sensitizer and the annihilator. Figure 7b shows the TTA-UC of these three compositions under sensitizer-matched conditions. We observed a well-resolved UC signal in DLPC among these three; however, the signal intensity in DMPC is reduced by 90% compared to DLPC. Although the signal was evident in DPPC 443 nm, the peak at 473 nm was broadened; it was difficult to quantitatively compare it to the other compositions. At ambient temperature (22 ± 1 °C), DLPC (C12) is expected to be more fluidic than DMPC and DPPC, thus facilitating sensitizer–annihilator collision. However, the difference observed here is attributed to background scatter from the DPPC liposomes (Figure S15). As they were found to be consistently larger than other liposome compositions, direct quantitative comparisons could not be made. However, it is clear that once the viscosity of the membranes increases, based on chain length-induced phase changes, the TTA-UC becomes much less intense.

Figure 7c illustrates the impact of membrane charge on TTA-UC. In this context, we prepared three different sets of liposomes comprising neutral (DOPC), negatively charged (DOPS), and positively charged (DOTAP) lipid membranes. As expected, DOPC, a neutral lipid, has the highest TTA-UC intensity when compared to DOPS, whereas DOTAP has the lowest TTA-UC intensity. At a physiological pH of 7.4, B2PI and perylene are neutral. However, BODIPY is formally zwitterionic, and this likely leads to electrostatic interaction between the COO^–^ of DOPS and the N^+^ of B2PI and between the (CH_3_)_3_N^+^ of DOTAP and the N^+^ of B2PI, which may impact the diffusion/collision of the sensitizer and the annihilator in each membrane; particularly in the case of DOPS, the fluidity of the membrane is identical to that of DOPC in Table 2, suggesting charge rather than fluidity is responsible for reducing the TTA-UC in these charged membranes.

### 2.4 TTA-UC at Plasma Membrane Mimetic Biomembranes

In considering the prospect of TTA-UC at biological membranes, we then explored whether TTA-UC could be observed at more typically biomimetic membranes. While it is difficult to exactly mimic the complexity of the eukaryotic cell membrane and its asymmetry in a liposome, TTA-UC was investigated using egg phosphatidylcholine total extract, that may partially mimic the eukaryotic membrane, since it comprises a natural composition of different alkyl chain lengths of different unsaturated and saturated alkyl chains but comprises L_D_ phase and is fluidic at room temperature.^62^ As can be seen in Figure 7d, the fluidic nature of egg PC is reflected in the measured D and the notably high TTA-UC intensity. Further extending to the higher mammalian plasma membrane (MPM) mimetic lipid compositions for the TTA-UC study, we chose a membrane composition containing 32% DOPC, 25% DOPE, 20% cholesterol, 15% SM, and 8% PE, which was coined “Nature’s Own” by Lentz et al.^63,64^ Since the MPM composition is also highly fluidic in nature (*D* = 7.89 ± 0.42 μm^2^ s^–1^),^65^ we observed a high UC intensity (Figure 7e). One of the reasons, we speculate, for the high intensity of TTA-UC in such complex membranes is the propensity of the sensitizer to localize in the *L*_d_ phase, which is prevalent in these compositions, and promotes the diffusional collision required for TTA-UC.

Liposomal TTA-UC systems offer enormous potential for implementing upconversion in biological systems, but to date, very few examples have been reported. As TTA-UC requires mutual collision between the sensitizer and the annihilator, important considerations are both the mutual affinity and orientation of the sensitizer and the annihilator within the membrane and the mobility of the sensitizer and annihilator pair, to ensure effective collision for Dexter energy transfer. Both are likely influenced by the composition and viscosity of the membrane. Here, we report the first organic liposomal TTA-UC system based on a BODIPY sensitizer using a perylene substituted charge transfer complex and a perylene annihilator. The doping of the constituents into liposomes was visualized at GUVs using confocal microscopy for the TTA-UC pairs within both single-component DOPC and phase-separated domains forming DOPC:SM:Chol GUVs. We observed that both sensitizers uniformly localized at the DOPC bilayer, whereas in DOPC:SM:Chol, the sensitizer localized into the liquid disordered phase. The upconversion spectra confirm that the emission is indeed from perylene while exciting the dyad sensitizer, and we observed that the BODIPY-perylene dyad has a long triplet lifetime, which is necessary for efficient bimolecular triplet–triplet energy transfer processes. Molecular dynamics simulations reveal why the process is so efficient in membranes, confirming that the sensitizer and annihilator co-locate within the hydrophobic core and form effective collision complexes within the membrane that persist for a time window that exceeds the TTA kinetics, from transient spectroscopy. We observe that the TTA-UC relative intensity is reduced at the phase-separated DOPC:SM:Chol GUV, and this is attributed to the observation that while the sensitizer is phase-localized, the annihilator perylene is not. Thus, the relative concentration of the annihilator and sensitizer is unbalanced by phase separation, thus reducing the TTA-UC relative intensity compared to the homogeneous membrane and revealing an important consideration where TTA-UC is implemented in complex liposome compositions or membranes.

On doping of B2P–perylene and B2PI–perylene pairs into large unilamellar vesicles comprising different membrane compositions, we observed a viscosity-dependent variation in the TTA-UC intensity. The intensity of TTA-UC was found to decrease exponentially according to DOPC > POPC > DLPC ≫ DMPC, attributed to the reduced collision frequency of the sensitizer and the annihilator in more viscous compositions. This conclusion is further substantiated by molecular dynamics simulations, which indicate that the rate of the sensitizer–annihilator complex formation decreases as the membrane’s conformational ordering and viscosity increase.

## 3 Conclusions

Herein, we present two BODIPY-perylene dyad sensitizers exhibiting TTA-UC with perylene in liposomes. This study demonstrates that TTA-UC can be effectively implemented in liposomal systems across the compositions and phases. It provides important new insights into what dictates the relative intensity of TTA-UC at liposomal membranes, the importance of the collision complex between the sensitizer and the annihilator, and that the influence of the membrane on facilitating this complex is critical. These new insights should be helpful in aiding the future design of TTA-UC in membranal systems, e.g., in drug delivery or to cellular systems, where it has tremendous potential for photoactivated drug delivery applications.

## 4 Experimental Section

### 4.1 Materials

Ph-BODIPY-2-perylene (B2P) and Ph-BODIPY-2-perylene-iodine (B2PI) were synthesized according to the procedure given in our recent publication.^4^ 1,2-Dioleoyl-*sn*-glycero-3-phosphocholine (DOPC) [purity (>99%)], 1,2-dimyristoyl-*sn*-glycero-3-phosphocholine (DMPC), 1-palmitoyl-2-oleoyl-glycero-3-phosphocholine (POPC), 1,2-dilauroyl-*sn*-glycero-3-phosphocholine (DLPC), 1,2-dipalmitoyl-*sn*-glycero-3-phosphocholine (DPPC), 1,2-dioleoyl-*sn*-glycero-3-phospho-l-serine (DOPS), 1,2-dioleoyl-3-trimethylammonium-propane (DOTAP), L-α-phosphatidylcholine (Egg PC), and sphingomyelin (SM, brain, porcine) were purchased from Avanti Polar Lipids (Alabama) and used without further purification. 1,2-Dioleoyl-*sn*-glycero-3-phosphoethanolamine-labeled ATTO655 (DOPE-ATTO655) was purchased from ATTO-TEC GmbH (Siegen, Germany). DiIC18(5) solid (1,1′-dioctadecyl-3,3,3′,3′-tetramethylindodicarbocyanine, 4-chlorobenzenesulfonate salt) (DiD) was purchased from ThermoFisher Scientific. Perylene, sodium sulfite, cholesterol, D-(+)-glucose, sucrose, agarose, 1,4-dioxane, and phosphate-buffered saline (PBS) tablets were purchased from Sigma-Aldrich (Wicklow, Ireland). Aqueous solutions were prepared using Milli-Q water (Millipore Corp., Bedford). The polydimethylsiloxane (PDMS) silicon elastomer was purchased from Dow Corning GmbH (Wiesbaden, Germany) and mixed following supplier instructions. Monodisperse polystyrene (PS) latex sphere with a diameter of 4.6 ± 0.4 μm was obtained from Bangs Laboratories Inc. (Fishers, IN).

### 4.2 Photophysical Steady-State Studies

Electronic absorption spectra were acquired on a Jasco V670 UV/vis NIR spectrophotometer using a quartz cuvette with a 1 cm path length. Fluorescence spectra were collected on a Varian Cary Eclipse fluorescence spectrofluorometer. All photophysical measurements were performed at room temperature (293 K).

The TTA-UC measurements were carried out in the same fluorescence spectrophotometer using an additional laser purchased from Edmund Optics with 532 nm excitation of 10 mW power with a 1 mm beam diameter. The TTA-UC measurements were performed in a fluorescence spectrophotometer by blocking the excitation line in the bioluminescence measurement mode. The upconverted emission in vesicles was recorded in the presence of 20 mM sodium sulfite as an oxygen scavenger. The upconverted emission was recorded across the range of 400–500 nm to avoid interference from the excitation source.

### 4.3 Photophysical Time-Resolved Studies

Luminescent lifetime data were acquired up to 10,000 counts using a time-correlated single photon counting (TCSPC) system by PicoQuant with a laser excitation source 450 nm. Measurements were performed in triplicate, and PicoQuant FluoFit software was used for data analysis and fitting.

Nanosecond time-resolved absorption (ns-TA) data were acquired using a custom-built setup reported earlier.^66^ The electronics and programming to record the difference absorption signal (for ns-TA) are developed by Pascher Instruments (Lund, Sweden). The samples were kept in 1 cm path length quartz-made inert cuvettes for all ns-TA measurements. The pump pulse energy was 0.12 ± 0.01 mJ for all ns-TA measurements.

### 4.4 Reconstitution of Sensitizers and Annihilators into Large Unilamellar Vesicles (LUVs)

Reconstitution of BODIPY-perylene dyads into LUVs was carried out using a hydration extrusion method. The sensitizers were mixed with lipid in a 5088:10:1 molar ratio of lipid:annihilator:sensitizer in chloroform in a 1.5 mL glass vial, and the mixture was dried under nitrogen flow, leading to the formation of a lipid film along the sides of the glass vial. Complete evaporation of the solvent was ensured by keeping it under vacuum for 60 min. The film was then hydrated with 1 mL of phosphate-buffered saline (PBS) at pH 7.4, and the film was suspended into the buffer by vortexing for 60 s. This solution was extruded through a 100 nm polycarbonate membrane typically 11 times to achieve uniform large unilamellar vesicles (LUVs) of around 100 nm diameter. The liposomes were dialyzed using a pur-A-lyzer kit.^67^ The diameter and homogeneity of the resulting liposomes were confirmed using dynamic light scattering (DLS) of the Malvern Zetasizer Ultra.

For TTA-UC studies in liposomes, 0.25 μM B2PI and 2.5 μM perylene were reconstituted into LUVs of different lipid compositions. The concentration was approximately a factor of 4 lower than the concentrations optimized previously in solution, but the ratio of 1:10 is maintained.^4^ In all cases, complete integration of the sensitizers and annihilators into the membrane was confirmed by analyzing the filters and filtrate for unbound materials.

### 4.5 Preparation of Giant Unilamellar Vesicles

Both single and ternary giant unilamellar vesicles (GUVs) were prepared by electroformation using the Vesicle Prep Pro (VPP) (Nanion Technologies, Munich, Germany).^53^ Single-phase GUVs comprised a DOPC lipid, and the ternary GUVs were prepared using DOPC, sphingomyelin (SM), and cholesterol in a 2:2:1 molar ratio to a final concentration of 5 mM; this ratio forms phase-separated domains in the GUV. The sensitizer and annihilator molecules were mixed with the lipid stock with a 5088:10:1 lipid:annihilator:sensitizer molar ratio. To identify the phase selectivity of the sensitizer in the vesicle, a lipophilic tracer dye DiD (DiIC18(5) solid (1,1′-dioctadecyl-3,3,3′,3′-tetramethylindodicarbocyanine, 4-chlorobenzenesulfonate salt)) was used to colabel the vesicle at a concentration of 0.1 mol % to lipid along with the sensitizer. The lipid-dye stock solution was drop-cast as several droplets of 1 μL onto a pair of conductive ITO slides, and the solvent was evaporated by purging with N_2_ and then stored in the vacuum for 45 min. ITO slides were carefully introduced into the chamber along with a 0.23 mM warm sucrose solution, and the slides were placed one above the other separated by an O-ring of 0.7 mm thickness greased on the conductive side of the ITO slides. The electroformation was started by applying an alternating current voltage of 0–3 V and 55 °C temperature raised for 5 min, and 3 V was continuously applied at 10 Hz frequency for 170 min at 55 °C followed by a fall time of 5 min. The vesicle solution was collected from the slides using an enlarged-sized pipet tip by cutting the edge and adding to the 0.23 mM glucose solution to maintain the osmolarity.

### 4.6 Confocal Microscopy of Giant Unilamellar Vesicles (GUVs)

Confocal fluorescence images of the GUVs were collected by using a Leica TSP inverted (DMi8) confocal microscope. The excitation line was selected from a white light laser used as an excitation source, and a 40×-oil immersion objective was used. Samples with B2P were excited at 514 nm, and the emission was collected at 630–640 nm. Similarly, the excitation and emission wavelengths (λ_ex_/λ_em_) for other dyes were as follows: 514/650–660 nm for B2PI and 633/665–700 nm for DiD. The GUVs were mixed with 0.5% agarose for imaging to impede the movement of the vesicles and imaged after 10 min of agarose addition.

### 4.7 PDMS Microcavity Array Preparation

20 μL of 0.1% of 4.61 μm PS spheres dispersed in ethanol was drop-cast over 1 cm × 1 cm pieces of hand-cleaved mica fixed on a glass slide. After the evaporation of ethanol, PDMS was mixed with a curing agent in a 10:1 ratio and was poured over the PS sphere arrays and dried at 90 °C for 60 min. The PS spheres were then removed from the PDMS substrate by sonicating in THF for 15 min, and the microcavity arrays of approximately 2 μm diameter were thus obtained. Complete evaporation of THF was ensured by drying the substrate overnight at room temperature. Critically, before lipid bilayer formation, the substrates were rendered hydrophilic by treatment with oxygen plasma for 5 min prior to aqueous filling; this ensured complete aqueous filling of the pores and a sufficiently hydrophilic interface to support the bilayers. Without this step, the bilayers are not stable. Following this, the microcavities were filled with buffer by sonication for 15 min in PBS.

### 4.8 Preparation of Microcavity Array-Supported Lipid Bilayers (MSLBs)

The DOPC lipid bilayer was spanned across aqueous-filled PDMS microcavities to form the microcavity-supported lipid bilayer. Initially, the lipid monolayer was transferred using the Langmuir–Blodgett (LB) technique at the air–water interface as reported previously,^50−52^ whereby a 1 mg/mL lipid solution in cholesterol was added to the water subphase of an LB trough (NIMA 102D) and the solvent was allowed to evaporate for about 10 min. The lipid monolayer was transferred to the microcavities at a 33 mN/m surface pressure. The liposome solution containing the sensitizer molecule was introduced to the microfluidic chamber of the PDMS substrate and incubated in the dark for about 90 min for the bilayer fusion to occur. Any residual liposomes were removed by washing with PBS, and this MSLB was then used for FLCS studies.

### 4.9 Fluorescence Lifetime Imaging (FLIM) and Correlation Spectroscopy (FLCS)

Fluorescence lifetime imaging (FLIM) and fluorescence lifetime correlation spectroscopy (FLCS) measurements on MSLB were performed using a MicroTime 200 system (PicoQuant GmbH, Germany) integrated with an FCS module, a dual single-photo avalanche diode (SPAD) detection unit, time-correlated single photon counting (TCSPC), and an inverted microscope model Olympus X1-71 with an Olympus UPlan SApo 60*x*/1.2 water immersion objective.

A 532 nm laser PicoTA from Toptica (Picoquant) with a pulse repetition rate of 20 MHz was used to excite the B2P probe and a 640 nm laser to excite the DOPE-ATTO655 probe embedded within the microcavity-supported lipid bilayer. The excitation light was directed to the sample through the objective lens by a 532/640 rpm dichroic mirror. The fluorescence emission was also collected through the same objective and filtered through the same dichroic mirror and a suitable interference filter. The detection volume in the axial direction was confined onto SPAD using a 50 μm pinhole. Further details are given in the Supporting Information.

### 4.10 Molecular Dynamics Simulations

#### 4.10.1 Simulation Systems and MD Protocol

We used three different models of lipid bilayers, each with a different lipid composition, which were prepared using the CHARMM-GUI Membrane Builder.^68^ These bilayers consisted of 120 DOPC, 132 DLPC, or 132 DMPC molecules, and each was embedded in a rectangular box measuring 6.5 nm × 6.5 nm × 7.6 nm. To solvate the bilayers, we added 5422, 5897, and 5889 TIP3 water molecules, respectively.^69^ To maintain a physiological ionic strength of 0.15 M, 24 K^+^ and 24 Cl^–^ ions were included in each of the systems. Subsequently, the standard CHARMM-GUI energy minimization and equilibration protocols were applied. The lipids were modeled using the CHARMM36m force field,^70^ while the force field parameters for B2P, B2PI, and perylene molecules were obtained from the CHARMM generalized force field (CGenFF)^71^ via the CHARMM-GUI server. The partial charges were determined by fitting to the electrostatic potential, which was calculated at the B3LYP-D3/def2svp level using Gaussian 16.^72^ Additionally, we refined the parameters for two dihedral angles in B2P and B2PI (θ_1_ and θ_2_ in Figure S15) by fitting to the rotational energy profiles computed at the B3LYP-D3/def2svp level. The equilibrium bond lengths and valence angles in the vicinity of the boron atom in B2P and B2PI were determined by adopting the values from the QM-optimized structures (B3LYP-D3/def2svp). Specifically, the bonds B–F, B–N, and N–C and angles F–B–F, F–B–N, B–N–C, and N–B–N were considered (see Figure S15). The same was done for the C–I bond length in the B2PI molecule. The complete sets of parameters used for B2P, B2PI, and perylene can be found in the attached file named “dyes.top”.

All energy minimizations and molecular dynamics simulations were carried out using Gromacs 2020.^73^ The simulations were performed in periodic boundary conditions and in the NPT ensemble with the temperature kept at 310.15 K using the Nose–Hoover thermostat^74^ with a coupling time of 1 ps, while the pressure was coupled semi-isotropically with a Parrinello–Rahman barostat at 1 bar with a coupling time of 5 ps.^75^ To calculate electrostatic interactions, the particle-mesh Ewald summation was applied with a real space cutoff equal to 1.2 nm and a Fourier grid spacing of 0.12 nm. van der Waals interactions were represented by the Lennard-Jones potential with a smooth cutoff with a switching radius of 1.0 nm and a cutoff radius of 1.2 nm. The P-LINCS algorithm^76^ was used to constrain the length of all bonds involving hydrogen atoms except the water molecules, for which SETTLE was used.^77^ The Verlet leapfrog algorithm was used to integrate the equations of motion with the time step of 2 fs.

#### 4.10.2 Spontaneous Membrane Binding and Localization in the Membrane

To examine the preferred localization of the studied sensitizer and annihilator molecules within a lipid bilayer, we introduced single molecules of B2P, B2PI, or perylene into the water phase of the pre-equilibrated DOPC system. Subsequently, each of the three systems thus prepared was subjected to MD simulation following the described protocol, with five independent replicas conducted to ensure sufficient sampling. After the spontaneous penetration of the molecules into the lipid phase (within tens of nanoseconds; see Movie S1), each replica was simulated for the next 1.2–1.6 μs. The distributions of molecules along the axis perpendicular to the membrane surface were computed after discarding the first 300 ns of each replica as equilibration.

#### 4.10.3 Free Energy Simulations for Sensitizer–Annihilator Binding within Lipid Bilayers

To study the sensitizer–annihilator interaction in the three lipid membranes (DOPC, DLPC, and DMPC), we determined how the system’s free energy changes as a function of the center-of-mass distance, *r*, between the perylene fragment of the B2P or B2PI and the annihilator perylene molecule (see the inset in Figure 5). To this end, we performed replica exchange umbrella sampling (REUS) simulations using the PLUMED 2.8 plugin^78^ coupled to Gromacs. To capture the formation of the sensitizer–annihilator collision complexes, the reaction coordinate was sampled in the range spanning from 0.3 nm (tightly bound state) (0.3 nm) up to 3.0 nm (fully dissociated state) in 28 uniformly distributed and 0.1 nm-separated REUS windows. The initial configurations for these windows were obtained from the additional unbiased 1.2-μs-long MD simulations in which the sensitizer and annihilator molecules were allowed to spontaneously bind to each other in the three lipid membranes considered. In each of the REUS windows, the system was simulated for 0.5 μs, using the harmonic potential with a force constant of 240 kcal/(mol·nm2) to restrain the system along the reaction coordinate *r*. The exchanges between neighboring windows were attempted every 2 ps, and the acceptance rate turned out to be ∼11%. The free energy profiles were determined from the last 450 ns of the thus-obtained trajectories using the standard weighted histogram analysis method (WHAM 2.0.9).^79^ Uncertainties were estimated using bootstrap error analysis, taking into account the correlation in the analyzed time series.

#### 4.10.4 Binding Kinetic Calculations

To calculate the rate constants, *k*_on_, for formation of the tightly bound B2PI/perylene complexes in DOPC and DMPC membranes, we first determined the binding constant *K* by integrating the Boltzmann distribution over the reaction coordinate *r*: , where *G*(*r*) is the MD-derived free energy profile, *V*_0_ is the standard volume (1661 Å^3^) corresponding to the standard concentration of 1 M, *R* is the upper distance limit defining the tightly bound complex (minimum I in Figure 5a), *k*_B_ is the Boltzmann constant, and *T* is the temperature. Next, the binding rate constant was computed as *k*_on_ = *Kk*_off_, where *k*_off_ is the dissociation rate constant. To determine *k*_off_, we estimated the average lifetime of the tightly bound B2PI/perylene complex by conducting 20 unbiased MD simulations, each initiated in the tightly bound state (*r* < 0.55). Then, we computed *k*_off_ as the inverse of this average lifetime.

## Supporting Information Available

Detailed description of materials and methods, steady-state spectroscopic data, and FLCS data (PDF)
Rapid assembly of B2P and B2PI with high affinity into a DOPC lipid bilayer (avi)
MD simulations of spontaneous binding/dissociation (avi)

## Supplementary Material

am4c00990_si_001.pdf

am4c00990_si_002.avi

am4c00990_si_003.avi

## Abbreviations

B2P: Ph-BODIPY-2-perylene
B2PI: Ph-BODIPY-2-perylene-iodine
LUV: large unilamellar vesicle
GUV: giant unilamellar vesicle
FLCS: fluorescence lifetime correlation spectroscopy
SM: sphingomyelin

## References

[ref1] Wu W.; Guo H.; Wu W.; Ji S.; Zhao J. Organic Triplet Sensitizer Library Derived from a Single Chromophore (BODIPY) with Long-Lived Triplet Excited State for Triplet–Triplet Annihilation Based Upconversion. J. Org. Chem. 2011, 76 (17), 7056–7064. 10.1021/jo200990y. 21786760

[ref2] Singh-Rachford T. N.; Castellano F. N. Photon Upconversion Based on Sensitized Triplet–Triplet Annihilation. Coord. Chem. Rev. 2010, 254 (21–22), 2560–2573. 10.1016/j.ccr.2010.01.003.

[ref3] Ye C.; Zhou L.; Wang X.; Liang Z. Photon Upconversion: From Two-Photon Absorption (TPA) to Triplet–Triplet Annihilation (TTA). Phys. Chem. Chem. Phys. 2016, 18 (16), 10818–10835. 10.1039/C5CP07296D. 26843136

[ref4] Arellano-Reyes R. A.; Prabhakaran A.; Sia R. C. E.; Guthmuller J.; Jha K. K.; Yang T.; Dietzek-Ivanšić B.; McKee V.; Keyes T. E. BODIPY-Perylene Charge Transfer Compounds; Sensitizers for Triplet-Triplet Annihilation Up-conversion. Chem. - Eur. J. 2023, 29 (24), e202300239 10.1002/chem.202300239. 36802283

[ref5] Jeyaseelan R.; Utikal M.; Daniliuc C. G.; Næsborg L. Photocyclization by a Triplet–Triplet Annihilation Upconversion Pair in Water – Avoiding UV-Light and Oxygen Removal. Chem. Sci. 2023, 14 (40), 11040–11044. 10.1039/D3SC03242F. 37860655 PMC10583691

[ref6] Parker C. A.; Hatchard C. G. Sensitized Anti-Stokes Delayed Fluorescence. Proc. Chem. Soc., London 1962, 386–387. 10.1039/PS9620000373.

[ref7] Naimovičius L.; Bharmoria P.; Moth-Poulsen K. Triplet–Triplet Annihilation Mediated Photon Upconversion Solar Energy Systems. Mater. Chem. Front 2023, 7 (12), 2297–2315. 10.1039/D3QM00069A. 37313216 PMC10259159

[ref8] Huang L.; Wu W.; Li Y.; Huang K.; Zeng L.; Lin W.; Han G. Highly Effective Near-Infrared Activating Triplet–Triplet Annihilation Upconversion for Photoredox Catalysis. J. Am. Chem. Soc. 2020, 142 (43), 18460–18470. 10.1021/jacs.0c06976. 33074671

[ref9] Liu Q.; Xu M.; Yang T.; Tian B.; Zhang X.; Li F. Highly Photostable Near-IR-Excitation Upconversion Nanocapsules Based on Triplet–Triplet Annihilation for in Vivo Bioimaging Application. ACS Appl. Mater. Interfaces 2018, 10 (12), 9883–9888. 10.1021/acsami.7b17929. 29425018

[ref10] Deka J.; Jha K. K.; Menon S.; Lal Krishna A. S.; Biswas R.; Raghunathan V. Microscopic Study of Resonant Third-Harmonic Generation from Amorphous Silicon Nanodisk Arrays. Opt. Lett. 2018, 43 (21), 5242 10.1364/OL.43.005242. 30382977

[ref11] Biswas R.; Dandu M.; Menon S.; Jha K. K..; Jyothsna K. M.; Majumdar K.; Majumdar K.; Raghunathan V. Third-Harmonic Generation in Multilayer Tin Diselenide under the Influence of Fabry-Perot Interference Effects. Opt Express 2019, 27 (20), 28855 10.1364/OE.27.028855. 31684630

[ref12] Vadrucci R.; Monguzzi A.; Saenz F.; Wilts B. D.; Simon Y. C.; Weder C. Nanodroplet-Containing Polymers for Efficient Low-Power Light Upconversion. Adv. Mater. 2017, 29 (41), 1702992 10.1002/adma.201702992. 28898468

[ref13] Askes S. H. C.; Bonnet S. Solving the Oxygen Sensitivity of Sensitized Photon Upconversion in Life Science Applications. Nat. Rev. Chem. 2018, 2 (12), 437–452. 10.1038/s41570-018-0057-z.

[ref14] Mattiello S.; Monguzzi A.; Pedrini J.; Sassi M.; Villa C.; Torrente Y.; Marotta R.; Meinardi F.; Beverina L. Self-Assembled Dual Dye-Doped Nanosized Micelles for High-Contrast Up-Conversion Bioimaging. Adv. Funct Mater. 2016, 26 (46), 8447–8454. 10.1002/adfm.201603303.

[ref15] Jha K. K.; Prabhakaran A.; Burke C. S.; Schulze M.; Schubert U. S.; Keyes T. E.; Jäger M.; Ivanšić B. D. Triplet-Triplet Annihilation Upconversion by Polymeric Sensitizers. J. Phys. Chem. C 2022, 126 (8), 4057 10.1021/acs.jpcc.1c09897.

[ref16] Xiao X.; Tian W.; Imran M.; Cao H.; Zhao J. Controlling the Triplet States and Their Application in External Stimuli-Responsive Triplet–Triplet-Annihilation Photon Upconversion: From the Perspective of Excited State Photochemistry. Chem. Soc. Rev. 2021, 50 (17), 9686–9714. 10.1039/D1CS00162K. 34263286

[ref17] Wohnhaas C.; Mailänder V.; Dröge M.; Filatov M. A.; Busko D.; Avlasevich Y.; Baluschev S.; Miteva T.; Landfester K.; Turshatov A. Triplet–T Riplet Annihilation Upconversion Based Nanocapsules for Bioimaging Under Excitation by Red and Deep-R Ed Light. Macromol. Biosci. 2013, 13 (10), 1422–1430. 10.1002/mabi.201300149. 23868857

[ref18] Dou Q.; Jiang L.; Kai D.; Owh C.; Loh X. J. Bioimaging and Biodetection Assisted with TTA-UC Materials. Drug Discovery Today 2017, 22 (9), 1400–1411. 10.1016/j.drudis.2017.04.003. 28433535

[ref19] Schloemer T.; Narayanan P.; Zhou Q.; Belliveau E.; Seitz M.; Congreve D. N. Nanoengineering Triplet–Triplet Annihilation Upconversion: From Materials to Real-World Applications. ACS Nano 2023, 17 (4), 3259–3288. 10.1021/acsnano.3c00543. 36800310

[ref20] Seo S. E.; Choe H.-S.; Cho H.; Kim H.; Kim J.-H.; Kwon O. S. Recent Advances in Materials for and Applications of Triplet–Triplet Annihilation-Based Upconversion. J. Mater. Chem. C 2022, 10 (12), 4483–4496. 10.1039/D1TC03551G.

[ref21] Kumar Jha K.; Prabhakaran A.; Cane Sia R.; Arellano Reyes R. A.; Kumar Sarangi N.; Yang T.; Kumar K.; Kupfer S.; Guthmuller J.; Keyes T. E.; Dietzek-Ivanšić B. Triplet Formation and Triplet-Triplet Annihilation Upconversion in Iodine Substituted Non-Orthogonal BODIPY-Perylene Dyads. ChemPhotoChem 2023, 7 (10), e202300073 10.1002/cptc.202300073.

[ref22] Huang L.; Kakadiaris E.; Vaneckova T.; Huang K.; Vaculovicova M.; Han G. Designing next Generation of Photon Upconversion: Recent Advances in Organic Triplet-Triplet Annihilation Upconversion Nanoparticles. Biomaterials 2019, 201, 77–86. 10.1016/j.biomaterials.2019.02.008. 30802685 PMC6467534

[ref23] Gray V.; Moth-Poulsen K.; Albinsson B.; Abrahamsson M. Towards Efficient Solid-State Triplet–Triplet Annihilation Based Photon Upconversion: Supramolecular, Macromolecular and Self-Assembled Systems. Coord. Chem. Rev. 2018, 362, 54–71. 10.1016/j.ccr.2018.02.011.

[ref24] Chen Q.; Liu Y.; Guo X.; Peng J.; Garakyaraghi S.; Papa C. M.; Castellano F. N.; Zhao D.; Ma Y. Energy Transfer Dynamics in Triplet–Triplet Annihilation Upconversion Using a Bichromophoric Heavy-Atom-Free Sensitizer. J. Phys. Chem. A 2018, 122 (33), 6673–6682. 10.1021/acs.jpca.8b05901. 30053373

[ref25] Pfund B.; Steffen D. M.; Schreier M. R.; Bertrams M.-S.; Ye C.; Börjesson K.; Wenger O. S.; Kerzig C. UV Light Generation and Challenging Photoreactions Enabled by Upconversion in Water. J. Am. Chem. Soc. 2020, 142 (23), 10468–10476. 10.1021/jacs.0c02835. 32412242

[ref26] Wang W.; Liu Q.; Zhan C.; Barhoumi A.; Yang T.; Wylie R. G.; Armstrong P. A.; Kohane D. S. Efficient Triplet–Triplet Annihilation-Based Upconversion for Nanoparticle Phototargeting. Nano Lett. 2015, 15 (10), 6332–6338. 10.1021/acs.nanolett.5b01325. 26158690

[ref27] Zhou Q.; Wirtz B. M.; Schloemer T. H.; Burroughs M. C.; Hu M.; Narayanan P.; Lyu J.; Gallegos A. O.; Layton C.; Mai D. J.; Congreve D. N. Spatially Controlled UV Light Generation at Depth Using Upconversion Micelles. Adv. Mater. 2023, 35 (46), e2301563 10.1002/adma.202301563. 37548335

[ref28] Kang J.-H.; Kim S.-H.; Fernandez-Nieves A.; Reichmanis E. Amplified Photon Upconversion by Photonic Shell of Cholesteric Liquid Crystals. J. Am. Chem. Soc. 2017, 139 (16), 5708–5711. 10.1021/jacs.7b01981. 28402658

[ref29] Zhang B.; Richards K. D.; Jones B. E.; Collins A. R.; Sanders R.; Needham S. R.; Qian P.; Mahadevegowda A.; Ducati C.; Botchway S. W.; Evans R. C. Ultra-Small Air-Stable Triplet-Triplet Annihilation Upconversion Nanoparticles for Anti-Stokes Time-Resolved Imaging. Angew. Chem., Int. Ed. 2023, 62 (47), e202308602 10.1002/anie.202308602. PMC10952532 37647167

[ref30] Yang H.; Guo S.; Jin B.; Luo Y.; Li X. Versatile, Stable, and Air-Tolerant Triplet–Triplet Annihilation Upconversion Block Copolymer Micelles. Polym. Chem. 2022, 13 (34), 4887–4894. 10.1039/D2PY00596D.

[ref31] Oddo A. M.; Mani T.; Kumar C. V. Micelles Embedded in Multiphasic Protein Hydrogel Enable Efficient and Air-Tolerant Triplet Fusion Upconversion with Heavy-Atom and Spin–Orbit Charge-Transfer Sensitizers. ACS Appl. Mater. Interfaces 2020, 12 (35), 39293–39303. 10.1021/acsami.0c11202. 32805935

[ref32] Lammers T.; Kiessling F.; Hennink W. E.; Storm G. Nanotheranostics and Image-Guided Drug Delivery: Current Concepts and Future Directions. Mol. Pharmaceutics 2010, 7 (6), 1899–1912. 10.1021/mp100228v. 20822168

[ref33] Chamundeeswari M.; Jeslin J.; Verma M. L. Nanocarriers for Drug Delivery Applications. Environ. Chem. Lett. 2019, 17 (2), 849–865. 10.1007/s10311-018-00841-1.

[ref34] Askes S. H. C.; Mora N. L.; Harkes R.; Koning R. I.; Koster B.; Schmidt T.; Kros A.; Bonnet S. Imaging the Lipid Bilayer of Giant Unilamellar Vesicles Using Red-to-Blue Light Upconversion. Chem. Commun. 2015, 51 (44), 9137–9140. 10.1039/C5CC02197A. 25940614

[ref35] Brion A.; Chaud J.; Klimezak M.; Bolze F.; Ohlmann L.; Léonard J.; Chassaing S.; Frisch B.; Kichler A.; Heurtault B.; Specht A. Photoactivatable Liposomes for Blue to Deep Red Light-Activated Surface Drug Release: Application to Controlled Delivery of the Antitumoral Drug Melphalan. Bioconjugate Chem. 2023, 34 (7), 1304–1315. 10.1021/acs.bioconjchem.3c00197. 37392184

[ref36] Askes S. H. C.; Brodie P.; Bruylants G.; Bonnet S. Temperature Dependence of Triplet–Triplet Annihilation Upconversion in Phospholipid Membranes. J. Phys. Chem. B 2017, 121 (4), 780–786. 10.1021/acs.jpcb.6b10039. 28059523 PMC5330659

[ref37] Askes S. H. C.; Bahreman A.; Bonnet S. Activation of a Photodissociative Ruthenium Complex by Triplet–Triplet Annihilation Upconversion in Liposomes. Angew. Chem., Int. Ed. 2014, 53 (4), 1029–1033. 10.1002/anie.201309389. 24339049

[ref38] Askes S. H. C.; Kloz M.; Bruylants G.; Kennis J. T. M.; Bonnet S. Triplet–Triplet Annihilation Upconversion Followed by FRET for the Red Light Activation of a Photodissociative Ruthenium Complex in Liposomes. Phys. Chem. Chem. Phys. 2015, 17 (41), 27380–27390. 10.1039/C5CP04352B. 26420663 PMC4642198

[ref39] Poznik M.; Faltermeier U.; Dick B.; König B. Light Upconverting Soft Particles: Triplet–Triplet Annihilation in the Phospholipid Bilayer of Self-Assembled Vesicles. RSC Adv. 2016, 6 (48), 41947–41950. 10.1039/C6RA07666A.

[ref40] O’Connor D.; Byrne A.; Dolan C.; Keyes T. E. Phase Partitioning, Solvent-Switchable BODIPY Probes for High Contrast Cellular Imaging and FCS. New J. Chem. 2018, 42 (5), 3671–3682. 10.1039/C7NJ04604A.

[ref41] Kiseleva N.; Filatov M. A.; Fischer J. C.; Kaiser M.; Jakoby M.; Busko D.; Howard I. A.; Richards B. S.; Turshatov A. BODIPY–Pyrene Donor–Acceptor Sensitizers for Triplet–Triplet Annihilation Upconversion: The Impact of the BODIPY-Core on Upconversion Efficiency. Phys. Chem. Chem. Phys. 2022, 24 (6), 3568–3578. 10.1039/D1CP05382E. 35084007

[ref42] Yang T.; Arellano-Reyes R. A.; Curley R. C.; Jha K. K.; Chettri A.; Keyes T. E.; Dietzek-Ivanšić B. In Cellulo Light-Induced Dynamics in a BODIPY-Perylene Dyad. Chem. - Eur. J. 2023, 29 (24), e202300224 10.1002/chem.202300224. 36807947

[ref43] Ly J. T.; Presley K. F.; Cooper T. M.; Baldwin L. A.; Dalton M. J.; Grusenmeyer T. A. Impact of Iodine Loading and Substitution Position on Intersystem Crossing Efficiency in a Series of Ten Methylated- *Meso* -Phenyl-BODIPY Dyes. Phys. Chem. Chem. Phys. 2021, 23 (21), 12033–12044. 10.1039/D0CP05904H. 33942042

[ref44] Tian Y.; Cheng Q.; Dang H.; Qian H.; Teng C.; Xie K.; Yan L. Amino Modified Iodinated BODIPY Photosensitizer for Highly Efficient NIR Imaging-Guided Photodynamic Therapy with Ultralow Dose. Dyes Pigm. 2021, 194, 109611 10.1016/j.dyepig.2021.109611.

[ref45] Bassan E.; Gualandi A.; Cozzi P. G.; Ceroni P. Design of BODIPY Dyes as Triplet Photosensitizers: Electronic Properties Tailored for Solar Energy Conversion, Photoredox Catalysis and Photodynamic Therapy. Chem. Sci. 2021, 12 (19), 6607–6628. 10.1039/D1SC00732G. 34040736 PMC8132938

[ref46] Roy S. G.; De P. Swelling Properties of Amino Acid Containing Cross-Linked Polymeric Organogels and Their Respective Polyelectrolytic Hydrogels with PH and Salt Responsive Property. Polymer 2014, 55 (21), 5425–5434. 10.1016/j.polymer.2014.08.072.

[ref47] Dols-Perez A.; Gramse G.; Calò A.; Gomila G.; Fumagalli L. Nanoscale Electric Polarizability of Ultrathin Biolayers on Insulating Substrates by Electrostatic Force Microscopy. Nanoscale 2015, 7 (43), 18327–18336. 10.1039/C5NR04983K. 26488226

[ref48] Przybylo M.; Sýkora J.; Humpolíčková J.; Benda A.; Zan A.; Hof M. Lipid Diffusion in Giant Unilamellar Vesicles Is More than 2 Times Faster than in Supported Phospholipid Bilayers under Identical Conditions. Langmuir 2006, 22 (22), 9096–9099. 10.1021/la061934p. 17042516

[ref49] Ruggiero E.; Alonso-de Castro S.; Habtemariam A.; Salassa L. Upconverting Nanoparticles for the near Infrared Photoactivation of Transition Metal Complexes: New Opportunities and Challenges in Medicinal Inorganic Photochemistry. Dalton Trans. 2016, 45 (33), 13012–13020. 10.1039/C6DT01428C. 27482656

[ref50] Sarangi N. K.; Prabhakaran A.; Keyes T. E. Multimodal Investigation into the Interaction of Quinacrine with Microcavity-Supported Lipid Bilayers. Langmuir 2022, 38, 6411 10.1021/acs.langmuir.2c00524. 35561255 PMC9134496

[ref51] Roy A.; Sarangi N. K.; Ghosh S.; Prabhakaran A.; Keyes T. E. Leaflet by Leaflet Synergistic Effects of Antimicrobial Peptides on Bacterial and Mammalian Membrane Models. J. Phys. Chem. Lett. 2023, 14 (16), 3920 10.1021/acs.jpclett.3c00119. 37075204 PMC10150393

[ref52] Sarangi N. K.; Prabhakaran A.; Roantree M.; Keyes T. E. Evaluation of the Passive Permeability of Antidepressants through Pore-Suspended Lipid Bilayer. Colloids Surf., B 2024, 234, 113688 10.1016/j.colsurfb.2023.113688. 38128360

[ref53] Jha K. K.; Prabhakaran A.; Spantzel L.; Sia R. C.; Pérez I.; Arellano-Reyes R. A.; Elmanova A.; Dasgupta A.; Eggeling C.; Börsch M.; Guthmuller J.; Presselt M.; Keyes T. E.; Dietzek-Ivanšić B. A BODIPY-Based Molecular Rotor in Giant Unilamellar Vesicles: A Case Study by Polarization-Resolved Time-Resolved Emission and Transient Absorption Spectroscopy. ChemPhotoChem. 2023, 7 (11), e202300091 10.1002/cptc.202300091.

[ref54] Filipe H. A. L.; Moreno M. J.; Róg T.; Vattulainen I.; Loura L. M. S. How To Tackle the Issues in Free Energy Simulations of Long Amphiphiles Interacting with Lipid Membranes: Convergence and Local Membrane Deformations. J. Phys. Chem. B 2014, 118 (13), 3572–3581. 10.1021/jp501622d. 24635540

[ref55] Janke J. J.; Bennett W. F. D.; Tieleman D. P. Oleic Acid Phase Behavior from Molecular Dynamics Simulations. Langmuir 2014, 30 (35), 10661–10667. 10.1021/la501962n. 25133680

[ref56] Sittig M.; Schmidt B.; Görls H.; Bocklitz T.; Wächtler M.; Zechel S.; Hager M. D.; Dietzek B. Fluorescence Upconversion by Triplet–Triplet Annihilation in All-Organic Poly(Methacrylate)-Terpolymers. Phys. Chem. Chem. Phys 2020, 22 (7), 4072–4079. 10.1039/D0CP00232A. 32031195

[ref57] Wang Z.; Sukhanov A. A.; Toffoletti A.; Sadiq F.; Zhao J.; Barbon A.; Voronkova V. K.; Dick B. Insights into the Efficient Intersystem Crossing of Bodipy-Anthracene Compact Dyads with Steady-State and Time-Resolved Optical/Magnetic Spectroscopies and Observation of the Delayed Fluorescence. J. Phys. Chem. C 2019, 123 (1), 265–274. 10.1021/acs.jpcc.8b10835.

[ref58] Filatov M. A.; Karuthedath S.; Polestshuk P. M.; Callaghan S.; Flanagan K. J.; Wiesner T.; Laquai F.; Senge M. O. BODIPY-Pyrene and Perylene Dyads as Heavy-Atom-Free Singlet Oxygen Sensitizers. ChemPhotoChem 2018, 2 (7), 606–615. 10.1002/cptc.201800020.

[ref59] Hirsch-Lerner D.; Barenholz Y. Probing DNA–Cationic Lipid Interactions with the Fluorophore Trimethylammonium Diphenyl-Hexatriene (TMADPH)1A Preliminary Report of This Study Was Presented at the “Artificial Self-Assembling Systems for Gene Transfer” Conferences of the Cambridge Healthtech Institute, September 28–29, 1995, Wakefield, MA, and November 17–18, 1996, Coronado, CA.1. Biochim. Biophys. Acta, Bioenerg. 1998, 1370 (1), 17–30. 10.1016/S0005-2736(97)00239-3. 9518536

[ref60] Gaber B. P.; Sheridan J. P. Kinetic and Thermodynamic Studies of the Fusion of Small Unilamellar Phospholipid Vesicles. Biochim. Biophys. Acta, Biomembr. 1982, 685 (1), 87–93. 10.1016/0005-2736(82)90038-4. 7059594

[ref61] Hernandez-Borrell J.; Keough K. M. W. Heteroacid Phosphatidylcholines with Different Amounts of Unsaturation Respond Differently to Cholesterol. Biochim. Biophys. Acta, Biomembr. 1993, 1153 (2), 277–282. 10.1016/0005-2736(93)90416-W. 8274498

[ref62] Sarangi N. K.; Shafaq-Zadah M.; Berselli G. B.; Robinson J.; Dransart E.; Di Cicco A.; Lévy D.; Johannes L.; Keyes T. E. Galectin-3 Binding to α _5_ β _1_ Integrin in Pore Suspended Biomembranes. J. Phys. Chem. B 2022, 126 (48), 10000–10017. 10.1021/acs.jpcb.2c05717. 36413808 PMC9743206

[ref63] Haque Md. E.; McIntosh T. J.; Lentz B. R. Influence of Lipid Composition on Physical Properties and PEG-Mediated Fusion of Curved and Uncurved Model Membrane Vesicles: “Nature’s Own” Fusogenic Lipid Bilayer. Biochemistry 2001, 40 (14), 4340–4348. 10.1021/bi002030k. 11284690

[ref64] Sarangi N. K.; Prabhakaran A.; Keyes T. E. Interaction of Miltefosine with Microcavity Supported Lipid Membrane: Biophysical Insights from Electrochemical Impedance Spectroscopy. Electroanalysis 2020, 32 (12), 2936 10.1002/elan.202060424.

[ref65] Gaul V.; Lopez S. G.; Lentz B. R.; Moran N.; Forster R. J.; Keyes T. E. The Lateral Diffusion and Fibrinogen Induced Clustering of Platelet Integrin α _IIb_ β _3_ Reconstituted into Physiologically Mimetic GUVs. Integrative Biology 2015, 7 (4), 402–411. 10.1039/C5IB00003C. 25720532

[ref66] Dura L.; Wächtler M.; Kupfer S.; Kübel J.; Ahrens J.; Höfler S.; Bröring M.; Dietzek B.; Beweries T. Photophysics of BODIPY Dyes as Readily-Designable Photosensitisers in Light-Driven Proton Reduction. Inorganics 2017, 5 (2), 21 10.3390/inorganics5020021.

[ref67] Radwan B.; Prabhakaran A.; Rocchetti S.; Matuszyk E.; Keyes T. E.; Baranska M. Uptake and Anti-Inflammatory Effects of Liposomal Astaxanthin on Endothelial Cells Tracked by Raman and Fluorescence Imaging. Microchim. Acta 2023, 190 (8), 332 10.1007/s00604-023-05888-8. PMC10374751 37500736

[ref68] Jo S.; Kim T.; Iyer V. G.; Im W. CHARMM-GUI: A Web-based Graphical User Interface for CHARMM. J. Comput. Chem. 2008, 29 (11), 1859–1865. 10.1002/jcc.20945. 18351591

[ref69] Jorgensen W. L.; Chandrasekhar J.; Madura J. D.; Impey R. W.; Klein M. L. Comparison of Simple Potential Functions for Simulating Liquid Water. J. Chem. Phys. 1983, 79 (2), 926–935. 10.1063/1.445869.

[ref70] Huang J.; Rauscher S.; Nawrocki G.; Ran T.; Feig M.; de Groot B. L.; Grubmüller H.; MacKerell A. D. CHARMM36m: An Improved Force Field for Folded and Intrinsically Disordered Proteins. Nat. Methods 2017, 14 (1), 71–73. 10.1038/nmeth.4067. 27819658 PMC5199616

[ref71] Vanommeslaeghe K.; Hatcher E.; Acharya C.; Kundu S.; Zhong S.; Shim J.; Darian E.; Guvench O.; Lopes P.; Vorobyov I.; Mackerell A. D. CHARMM General Force Field: A Force Field for Drug-like Molecules Compatible with the CHARMM All-atom Additive Biological Force Fields. J. Comput. Chem. 2010, 31 (4), 671–690. 10.1002/jcc.21367. 19575467 PMC2888302

[ref72] Frisch M. J.; Trucks G. W.; Schlegel H. B.; Scuseria G. E.; Robb M. A.; Cheeseman J. R.; Scalmani G.; Barone V.; Petersson G. A.; Nakatsuji H.; Li X.; Caricato M.; Marenich A. V.; Bloino J.; Janesko B. G.; Gomperts R.; Mennucci B.; Hratchian H. P.; Ortiz J. V.; Izmaylov A. F.; Sonnenberg J. L.; Williams-Young D.; Ding F.; Lipparini F.; Egidi F.; Goings J.; Peng B.; Petrone A.; Henderson T.; Ranasinghe D.; Zakrzewski V. G.; Gao J.; Rega N.; Zheng G.; Liang W.; Hada M.; Ehara M.; Toyota K.; Fukuda R.; Hasegawa J.; Ishida M.; Nakajima T.; Honda Y.; Kitao O.; Nakai H.; Vreven T.; Throssell K.; Montgomery Jr J. A.; Peralta J. E.; Ogliaro F.; Bearpark M. J.; Heyd J. J.; Brothers E. N.; Kudin K. N.; Staroverov V. N.; Keith T. A.; Kobayashi R.; Normand J.; Raghavachari K.; Rendell A. P.; Burant J. C.; Iyengar S. S.; Tomasi J.; Cossi M.; Millam J. M.; Klene M.; Adamo C.; Cammi R.; Ochterski J. W.; Martin R. L.; Morokuma K.; Farkas O.; Foresman J. B.; Fox D. J. Gaussian 16, Revision C.01.; Gaussian, Inc.: Wallingford CT, 2016.

[ref73] Abraham M. J.; Murtola T.; Schulz R.; Páll S.; Smith J. C.; Hess B.; Lindahl E. GROMACS: High Performance Molecular Simulations through Multi-Level Parallelism from Laptops to Supercomputers. SoftwareX 2015, 1–2, 19–25. 10.1016/j.softx.2015.06.001.

[ref74] Nosé S. A Unified Formulation of the Constant Temperature Molecular Dynamics Methods. J. Chem. Phys. 1984, 81 (1), 511–519. 10.1063/1.447334.

[ref75] Martoňák R.; Laio A.; Parrinello M. Predicting Crystal Structures: The Parrinello-Rahman Method Revisited. Phys. Rev. Lett. 2003, 90 (7), 075503 10.1103/PhysRevLett.90.075503. 12633242

[ref76] Hess B. P-LINCS: A Parallel Linear Constraint Solver for Molecular Simulation. J. Chem. Theory Comput. 2008, 4 (1), 116–122. 10.1021/ct700200b. 26619985

[ref77] Miyamoto S.; Kollman P. A. Settle: An Analytical Version of the SHAKE and RATTLE Algorithm for Rigid Water Models. J. Comput. Chem. 1992, 13 (8), 952–962. 10.1002/jcc.540130805.

[ref78] Tribello G. A.; Bonomi M.; Branduardi D.; Camilloni C.; Bussi G. PLUMED 2: New Feathers for an Old Bird. Comput. Phys. Commun. 2014, 185 (2), 604–613. 10.1016/j.cpc.2013.09.018.

[ref79] Kumar S.; Rosenberg J. M.; Bouzida D.; Swendsen R. H.; Kollman P. A. THE Weighted Histogram Analysis Method for Free-energy Calculations on Biomolecules. I. The Method. J. Comput. Chem. 1992, 13 (8), 1011–1021. 10.1002/jcc.540130812.

