# Supplementary material for: Triplet–Triplet Annihilation Upconverting Liposomes: Mechanistic Insights into the Role of Membranes in Two-Dimensional TTA-UC
Source: ACS Appl Mater Interfaces. 2024 May 22;16(22):29324–37. doi: 10.1021/acsami.4c00990 (PMC11163426; doi:10.1021/acsami.4c00990)
Supplement: Supplementary file 1 — am4c00990_si_001.pdf [file am4c00990_si_001.pdf]

Supporting Information

Triplet-Triplet Annihilation Upconverting Liposomes; Mechanistic Insights into the Role of Membrane in Two-Dimensional TTA-UC

Amrutha Prabhakaran,[#a] Keshav Kumar Jha,[#b] Rengel Cane E. Sia,[c] Ruben Arturo Arellano Reyes,[a] Nirod Kumar Sarangi,[a] Mateusz Kogut,[d] Julien Guthmuller,[c] Jacek Czub,[d] Benjamin Dietzek-Ivanšić[b] and Tia E. Keyes*[a]

[#]A.P and K.K.J. contributed equally to this paper.

[a]*School of Chemical Sciences and National Centre for Sensor Research, Dublin City University, Dublin 9, Ireland.*

[b]*Research Department Functional Interfaces, Leibniz Institute of Photonic Technology Jena, Jena 07745, Germany; Institute of Physical Chemistry and Abbe Center of Photonics, Friedrich Schiller University Jena, Jena 07743, Germany.*

[c]*Institute of Physics and Applied Computer Science, Faculty of Applied Physics and Mathematics, Gdańsk University of Technology, Narutowicza 11/12, 80233 Gdańsk, Poland.*

[d]*Department of Physical Chemistry, Gdańsk University of Technology, Narutowicza 11/12, 80233 Gdańsk, Poland.*

*Corresponding author

Email: tia.keyes@dcu.ie

Contents

1. Fluorescence lifetime imaging (FLIM) and correlation spectroscopy (FLCS) 1
2. Steady State Spectroscopy 3
3. Study on Lipid Bilayer Membranes 4
4. References 11

1. Fluorescence lifetime imaging (FLIM) and correlation spectroscopy (FLCS)

In FLCS, the diffusing fluorophore emission through the detection volume is analyzed by calculating the auto-correlation curve which measures the self-similarity of the signal as a function of time as defined below.

$$G(\tau) = \frac{(\langle \partial I(t) \rangle \langle \partial I(t+\tau) \rangle)}{\langle I(t) \rangle^2}$$ ....Equation 1

where ⟨⟩ denotes the time average, and ⟨∂I(t)⟩ and ⟨∂I(t+τ)⟩ are the fluorescent intensity fluctuations around the mean value at time, t and t+τ respectively, where τ is the lag time. The FLCS autocorrelation data were fitted to a 2D diffusion model using Equation 2:

$$G(\tau) = \left[\frac{1}{N}\right]\left[\frac{1}{1 + \left(\frac{\tau}{\tau_D}\right)^{\alpha}}\right]$$ ....Equation 2

where G(τ) is the autocorrelation function of fluorescence fluctuations, N is the average number of diffusing fluorophores in the effective volume, τ is the delay time, τ_D is the diffusion time of the molecules across the confocal volume, α is the anomalous parameter, and c is the contribution of the diffusing species. ACFs were fitted using the two-dimensional model of diffusion to determine the diffusion time and the diffusion coefficient was calculated using Equation 3:

$$D = \frac{\omega^2}{4\tau_D}$$ ....Equation 3

where D is the diffusion coefficient and ω is the $1/e^2$ radius of the confocal volume. ω was measured using ATTO-532/ATTO-655 (ATTO TEC, GmbH) dye solution of known diffusion coefficient at 20 °C in water. All measurements were performed with a dye concentration of 5 nM.

The fluorescence lifetime images were also taken in the same MicroTime 200 system. Each sample was acquired for 360 s with a 512 x 512 resolution. Data was analysed using PicoQuant Symphotime software.

3. Steady State Spectroscopy

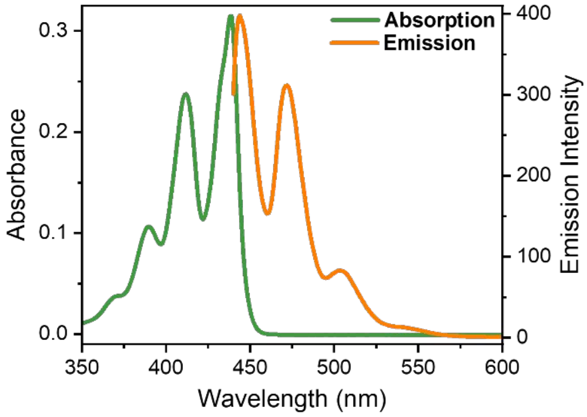

Figure S1: *Absorption (green line) and emission spectra (orange line) of 25 µM perylene in chloroform at emission slit widths of 2.5 nm respectively. Emission spectra was recorded by exciting the sample at 438 nm.*

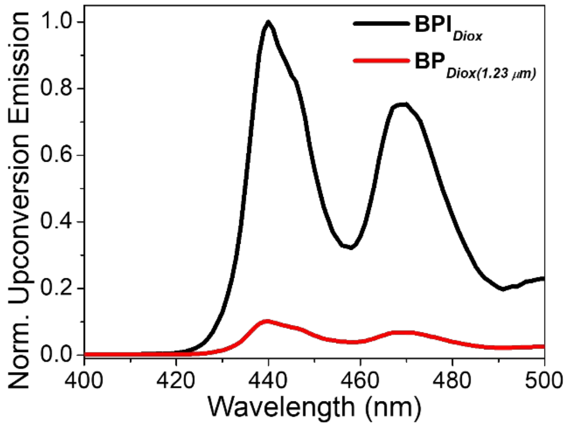

Figure S2: *Upconversion emission from BPI_{Diox} (black) and $BP_{Diox\ (1.23\ \mu M)}$ (red); both spectra are normalized by the emission peak from BPI_{Diox} sample. The samples were excited with 532 nm laser and the emission was collected with 2.5 nm slit width. Samples were deaerated by purging N_2. BPI_{Diox}: 1 µM B2PI and 10 µM perylene in dioxane, $BP_{Diox\ (1.23\ \mu M)}$: 1.23 µM B2PI and 10 µM perylene in dioxane. As can be seen in Figure S1, the emission of perylene extends to approximately 560 nm but spectra are cut off at 500 nm to exclude the excitation laser.*

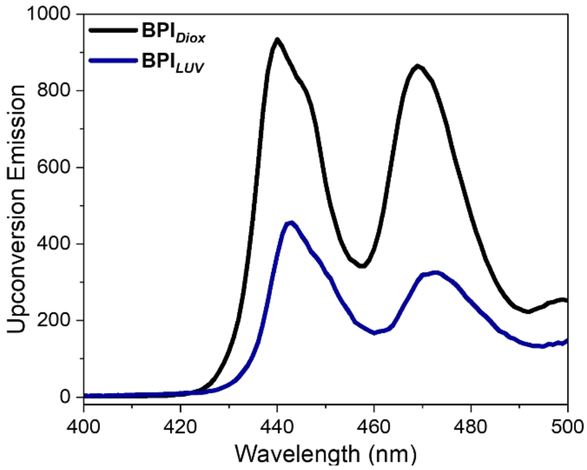

Figure S3: *Upconversion emission from BPI_{Diox} (black) and BPI_{LUV} (blue). The samples were excited with 532 nm laser and the emission was collected with 2.5 nm slit width. BPI_{Diox} was deaerated by purging N_2 and BPI_{LUV} was deaerated using 20*

mM sodium sulfite. BPI_{Diox}: 1 µM B2PI and 10 µM perylene in dioxane, BPI_{LUV}: 0.25 µM B2PI and 2.5 µM perylene in DOPC LUV. The small rise in the baseline above

4. Study on Lipid Bilayer Membranes

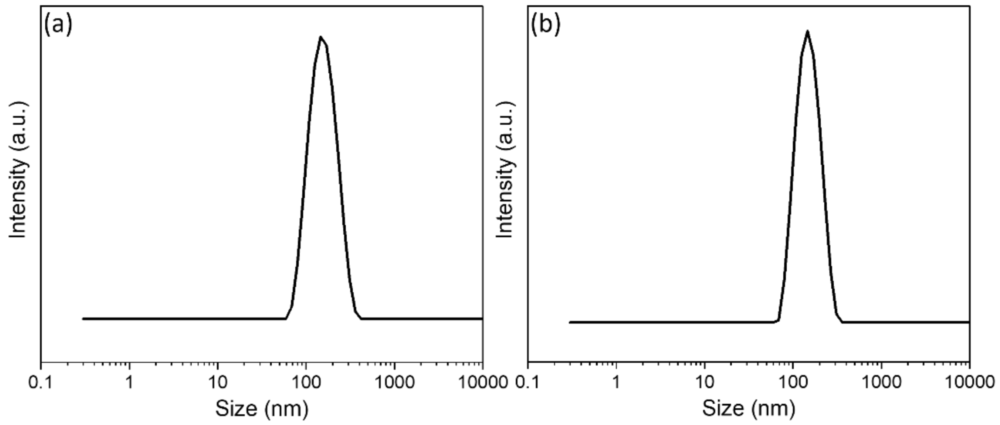

Figure S4: Dynamic light scattering spectra of DOPC liposomes containing 0.25 µM of each (a) B2P and (b) B2PI in PBS of pH 7.4. The average hydrodynamic radius is 140 nm.

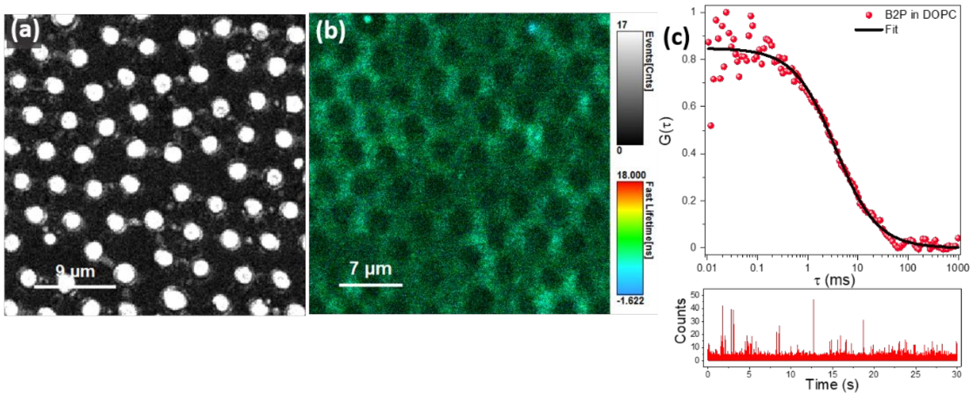

Figure S5: (a) Reflectance and (b) fluorescence lifetime images of microcavity supported lipid bilayer of DOPC labelled with B2P. (c) Representative normalised FLCS autocorrelation function measured over a single cavity and bottom panel shows the corresponding intensity-time trace.

Due to diffraction limit of optical microscope, visualizing the LUVs using a confocal microscope is difficult. Herein, we employed our previously established PDMS microcavity 2D array platform which is suitable for studying lipid-protein and lipid-drug interactions using confocal microscopy. The rationale behind using a pore suspended bilayer over PDMS microcavity array is to render a highly fluidic bilayer akin to GUV. Furthermore, in comparison to GUV, PDMS based suspended planar lipid bilayer system is more amenable for 2D surface sensitive techniques such as fluorescence imaging and correlation spectroscopy, protein reconstitution, control over its asymmetry across the leaflet, minimum sample volume in the microfluidic chamber to name a few.[1] The PDMS based microcavity array was prepared by soft lithographic as detailed in experimental methods which yields a close packed ordered array with dimensions ranging from µm to mm. Herein, to visualize the bilayer using FLIM, B2P sensitizer (0.01 mol%) was doped only to the upper leaflet of DOPC bilayer during the preparation of large unilamellar vesicles of size ~140 nm (cf. Figure S2) and fused over the LB transferred DOPC monolayer spanning the aqueous filled PDMS array. Figure S3a illustrates

the reflectance image of the buffer filled cavity arrays, where the white circular regions are indicative of the reflectance from the filled buffer originated from the refractive index mismatch between the PDMS and buffer. Typically, reflectance image was acquired first in order to assess whether the cavities are buffer filled. At instances, where the cavities are not occasionally buffer filled, the bilayer did not form due to the lack of lipid monolayer support, as indicated by arrow in Figure S4a and b. Occasional unfilled pores are evident in PDMS arrays because of the larger size of these pores (~2 μm diameter) and the hydrophobicity of the substrate that can sometimes mitigate against aqueous filling. The corresponding fluorescence lifetime image is shown in **Error! Reference source not found.**b in the identical regimes as that of Figure S3a. When combined both reflectance and FLIM image, it can be concluded that the bilayer is successfully spanned over the buffer filled cavity array. The image acquisition was carried out each time by observing the maximum brightness of the fluorophore (B2P) that was selectively doped in the outer membrane leaflet, and selectively positioning the confocal plane just over the bilayer plane. Due to such large cavity size, it allowed us to measure the point fluorescence correlation spectroscopy in lifetime mode by selectively focusing the observation/detection volume at the centre of such cavities that spanned the bilayer. At least 40-50 independent FLCS point measurements were typically acquired for each sample. **Error! Reference source not found.**S3c bottom panel shows a typical intensity-time trace originated when B2P diffuses in and out of the confocal volume. The fluorescence spikes are indicative of single B2P molecule diffusing translationally across the DOPC membrane in 2D. The ACF from FLCS was shown in **Error! Reference source not found.**S3c top panel as G(τ) versus lag time (τ) (red ball symbol). The solid line represents the fit using 2D diffusion model equation defined in equation S1. From the fit, the transit time (τ_D) was evaluated and accordingly the diffusion coefficient was found to be $8.6 \pm 0.4 \mu m^2 s^{-1}$, according to the equation S2. The observed D value is comparable to the commercial ATTO based lipidic probe, where the diffusivity was $9.6 \pm 0.5\ \mu m^2 s^{-1}$ (cf. Figure S4). Additionally, the anomalous coefficient (α) was found to be 1.01 indicating B2P diffusion was Brownian. The fluorescence lifetime of B2P from the MSLB is provided in Table S1 and the analysis of decay was shown in Figure S5. The overall result from both FLIM and FLCS measurements shows that B2P can not only suitably doped within the lipidic environment but also retains it optimal Brownian diffusion, within the dielectric lipidic environment, and established the array platform could be suitable model system for TTA-UC based studies.

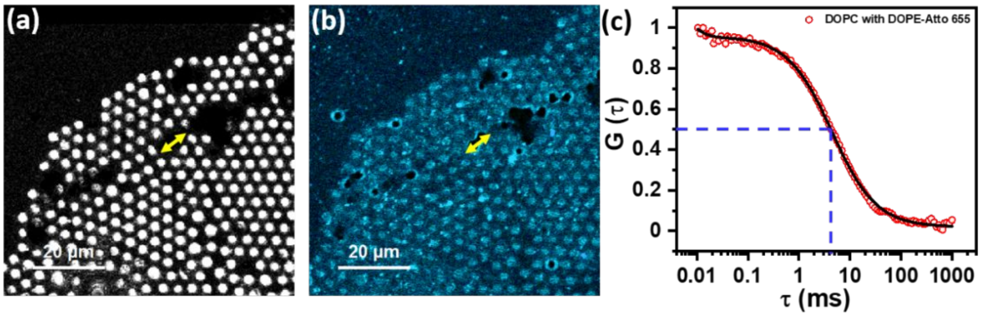

Figure S6: (a) Reflectance image and (b) Fluorescence Lifetime Image of DOPC lipid spanning cavities labelled with DOPE-Atto-655. (c) Normalized FLCS autocorrelation curve measured over a single cavity spanned with DOPC bilayer labelled with DOPE-Atto-655. Solid black line shows the fitted data.

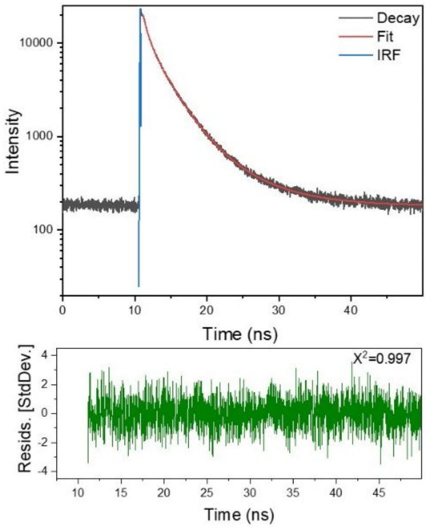

Figure S7: Time correlated single photon counting trace of B2P in DOPC spanned MSLB recorded extracted from FLCS data.

Table S1: Fluorescence lifetimes of B2P in dioxane, chloroform and DOPC MSLB measured over the cavity.

Sample	τ_1 (ns)	τ_2 (ns)	A_1 (kCnts)	A_2 (kCnts)
B2P in DOPC	2.81±0.05	6.8±0.3	11.9±0.06	7.9±0.12
B2P in Dioxane	1.79		6235.6	
B2P in Chloroform	2.76	1.24	855.1	5271

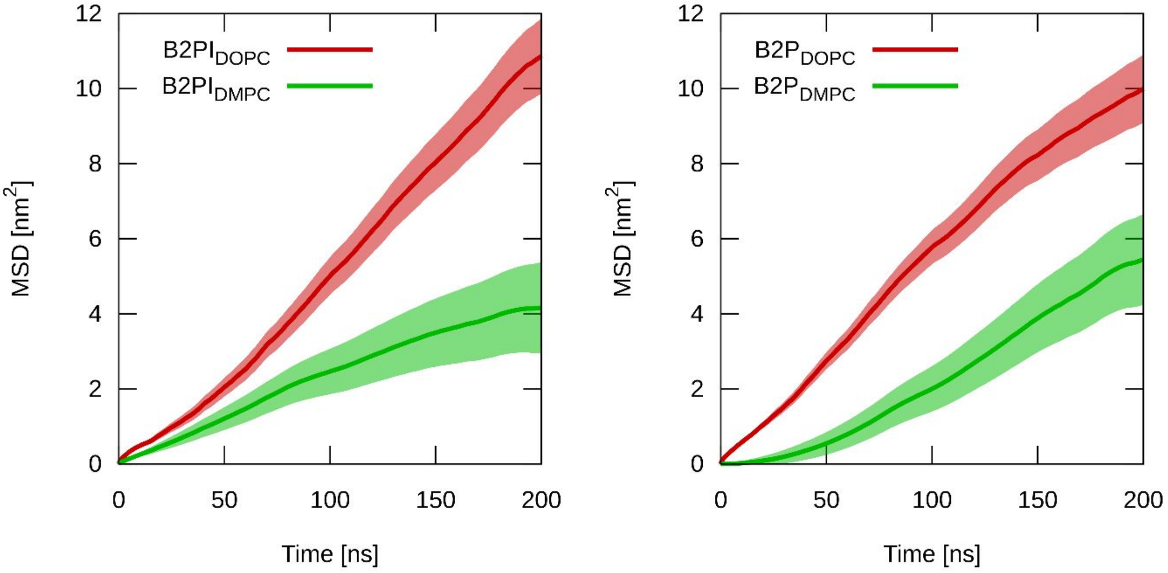

Figure S8: Time-dependence of the mean square displacement (MSD) of B2P and B2PI molecules in DOPC and DMPC lipid bilayers, computed from 5-µs-long MD trajectory data. To ensure statistical significance, we averaged the MSD curves over all continuous 200 ns intervals.

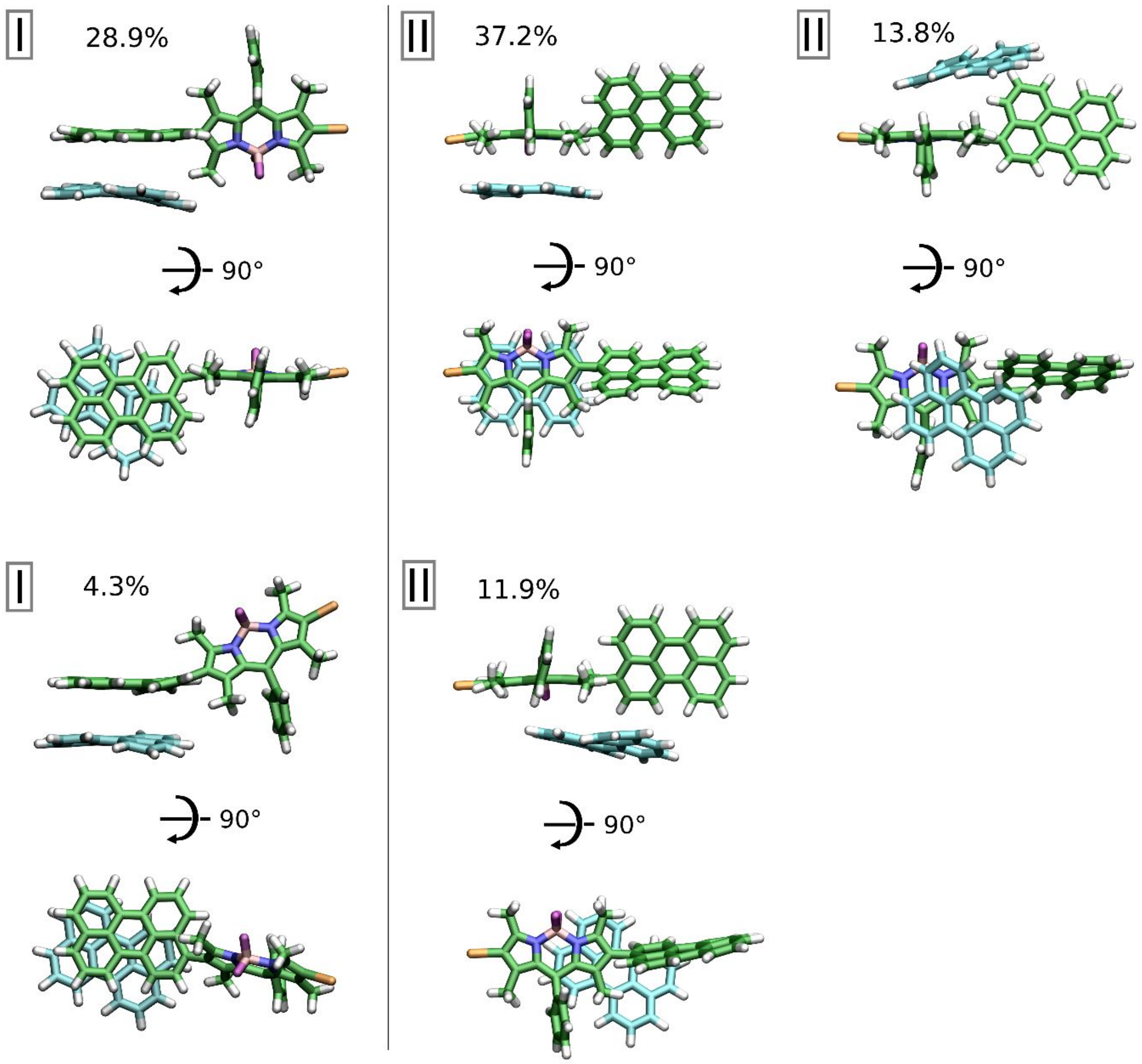

Figure S9: Depiction of B2PI/perylene collision complexes identified in our MD simulations including their respective populations in the bound state defined by the center-of-mass distance r<1.5 nm. For the sake of clarity, the B2PI and perylene carbon atoms are shown in green and cyan, respectively. Complexes of type I and II correspond to the two minima observed in the free energy profile (Figure 5).

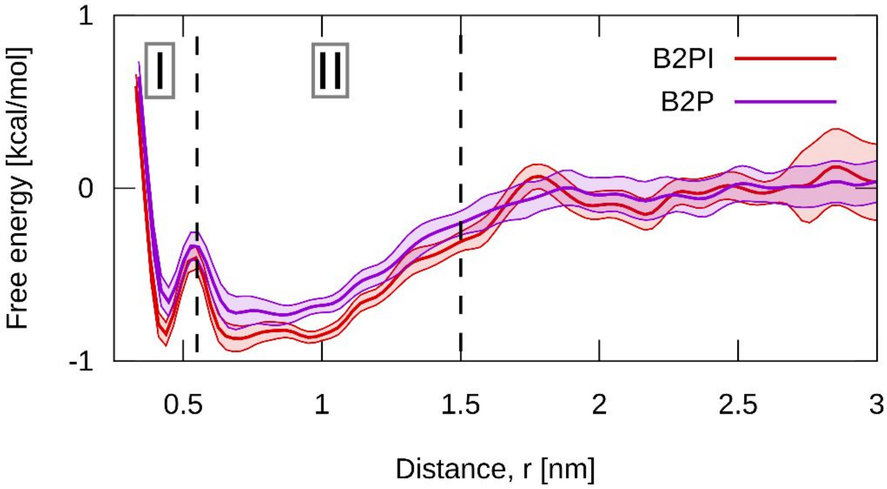

Figure S10: Comparison of the free energy profiles for the interaction of perylene with B2P (purple) and B2PI (red) in a DOPC lipid bilayer.

Protocol for the preparation of giant unilamellar vesicles (GUV)

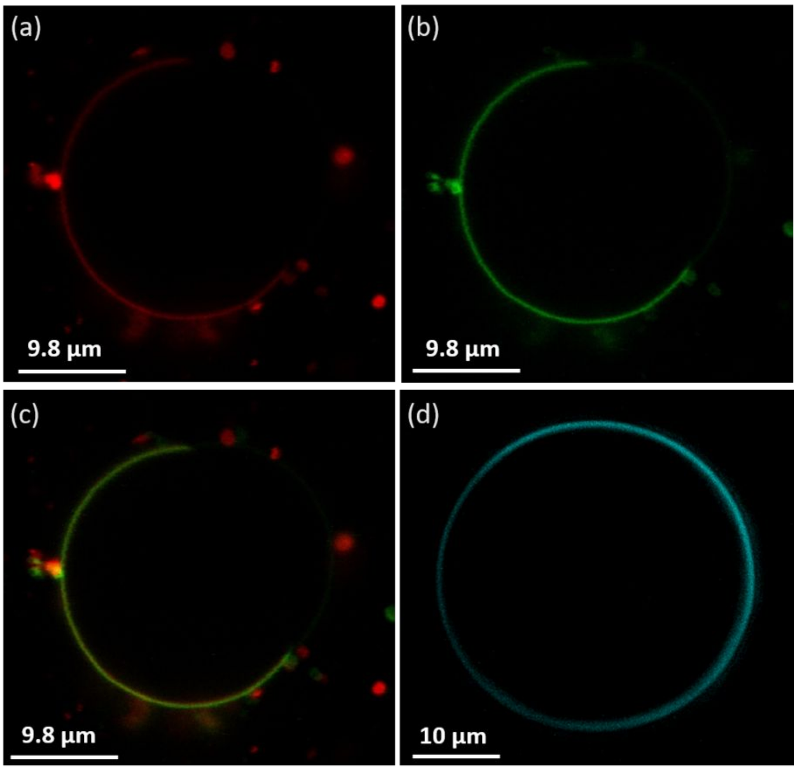

Figure S11: Confocal fluorescent imaging of phase-separated GUVs DOPC/BSM/Chol (2:2:1) mol%. GUV labelled with (a) DiD, (b) B2P, (c) overlay image, and (d) perylene. For B2P, $\lambda_{ex}/\lambda_{em}$=514/600-650 nm for DiD, $\lambda_{ex}/\lambda_{em}$=640/665-700 nm and for perylene, $\lambda_{ex}/\lambda_{em}$=405/440-500 nm.

The vesicles were co-labelled with B2P and DiD in GUVs composed of DOPC:SM:Chol. DiD, associates selectively with liquid disordered phase (L_d). Figure S9 shows the fluorescence images of the GUVs where the B2P is distributed inhomogenously in the ternary GUV co-

localising with the DiD tracer confirming it selectively partitions to the L_d phase. Similarly, it was also confirmed that B2PI also localised to L_d phase.

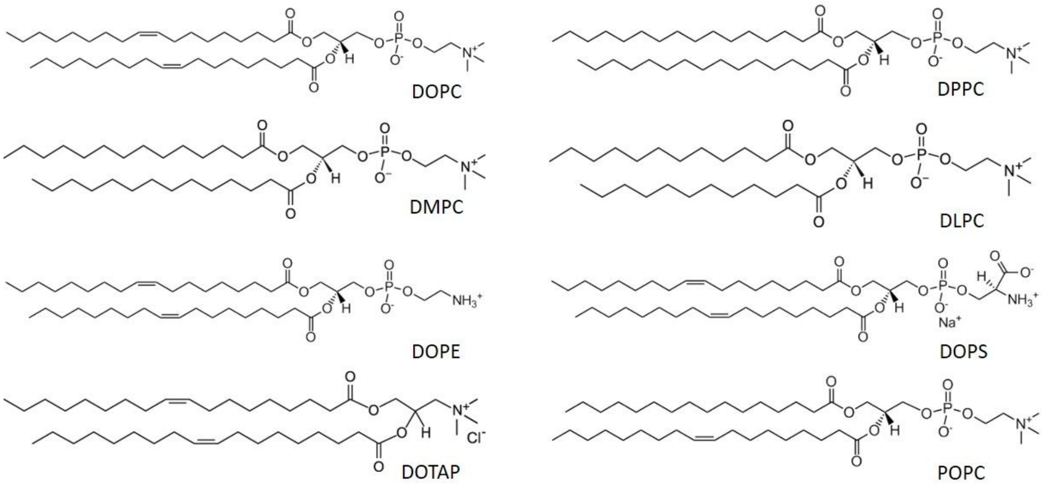

Figure S12: Chemical structures of different phospholipids used in this work.

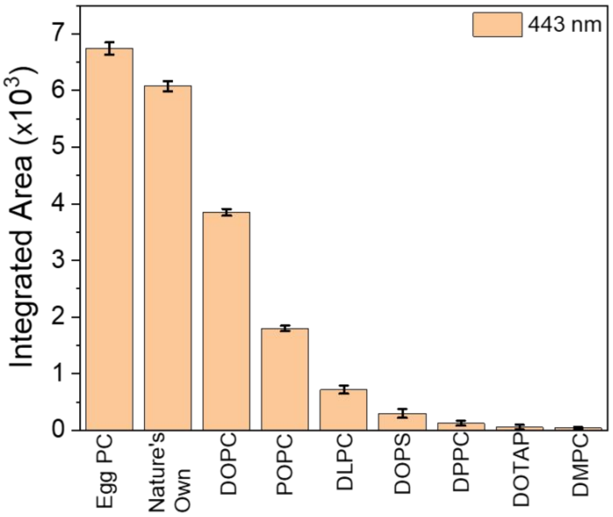

Figure S13: Comparison bar diagram of integrated mathematical area of 443 nm peak in different liposomes containing 0.25 µM B2PI and 2.5 µM perylene.

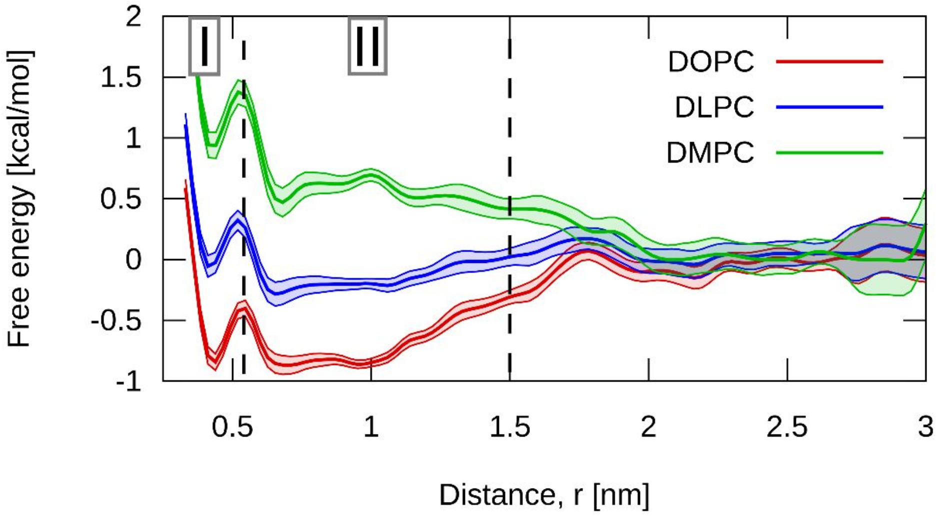

Figure S14: Influence of the lipid acyl chain type on the free energy profile for B2PI-perylene interaction within a lipid bilayer.

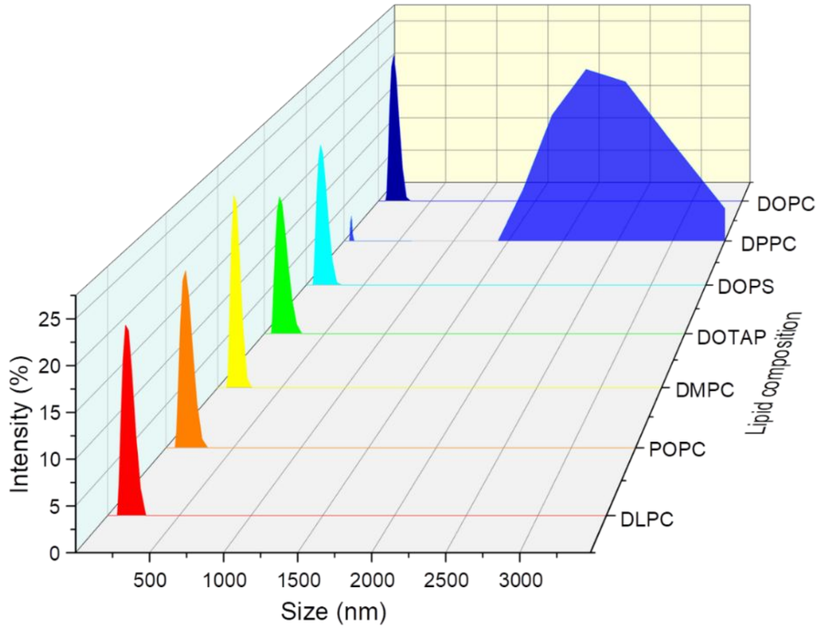

Figure S15: Dynamic light scattering data of different LUVs containing 0.25 µM B2PI and 2.5 µM perylene.

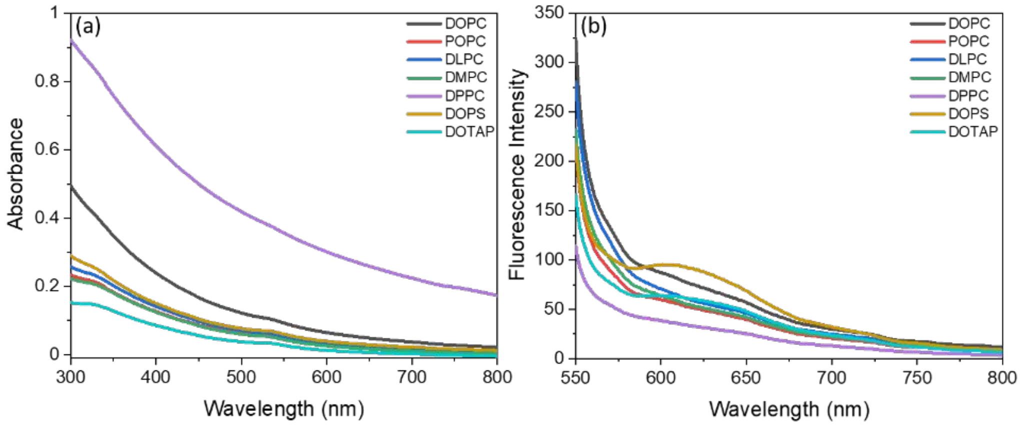

Figure S16:(a) Absorption and (b) emission spectra of 0.25 µM B2PI in different LUVs.

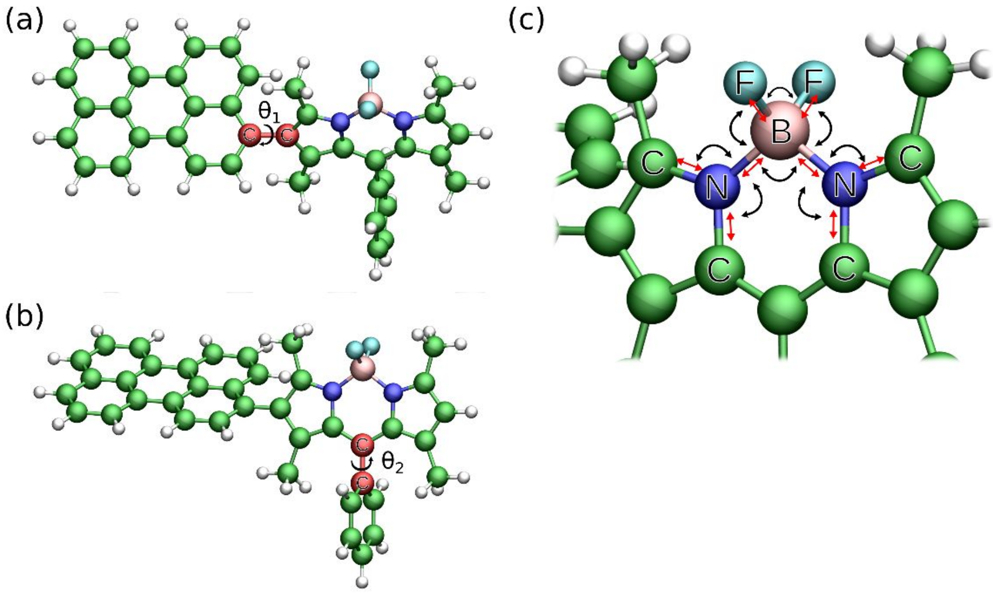

Figure S17: Bonded force-field terms in B2P and B2PI that were subject to refinement based on QM calculations: θ1 dihedral angle (A), θ2 dihedral angle (B), and bond lengths and valence angles in the vicinity of the boron atom (C). For numerical values see the attached file named 'dyes.top'.

5. References

(1) Berselli, G. B.; Sarangi, N. K.; Gimenez, A. V.; Murphy, P. V.; Keyes, T. E. Microcavity Array Supported Lipid Bilayer Models of Ganglioside – Influenza Hemagglutinin 1 Binding. *Chemical Communications* **2020**, *56* (76), 11251–11254. https://doi.org/10.1039/D0CC04276E.
